# Real-world analysis of depression-related adverse events associated with the adsorbed anthrax vaccine: integrating pharmacovigilance signals, machine learning risk prediction, and transcriptomic mechanisms

**DOI:** 10.1186/s40249-026-01483-0

**Published:** 2026-08-10

**Authors:** Guangwei Qing, Yimei Zhao, Jian Yang, Gang Wang

**Affiliations:** Beijing Key Laboratory of Intelligent Drug Research and Development for Mental Disorders; National Clinical Research Center for Mental Disorders; National Center for Mental Disorders; Beijing Anding Hospital, Capital Medical University, Beijing, China

**Keywords:** Adsorbed anthrax vaccine, Depression, Adverse event, Vaccine Adverse Event Reporting System, Food and Drug Administration Adverse Event Reporting System, Disproportionality analysis, Machine learning, Transcriptomic analysis

## Abstract

**Background:**

Anthrax remains a life-threatening zoonotic disease in resource-limited settings. Adsorbed anthrax vaccine (AVA, BioThrax) is the only United States-authorized preexposure prophylaxis for high-risk *Bacillus anthracis* exposure. Although AVA safety has been described, depression-related adverse events (AEs) remain insufficiently characterized. This study evaluated real-world depression-related safety signals associated with AVA and anthrax-related antibacterial agents.

**Methods:**

AVA-related AE reports were extracted from the Vaccine Adverse Event Reporting System (VAERS), and depression-related AE reports for seven anthrax-related antibacterial agents were retrieved from the FDA Adverse Event Reporting System (FAERS). AEs were coded using Medical Dictionary for Regulatory Activities (MedDRA). Disproportionality analyses were performed using four standard signal-detection methods, with sensitivity analysis excluding concurrent vaccination. Seven complementary machine learning algorithms, covering regression, tree-based, boosting, kernel, instance-based, and probabilistic methods, explored predictors, with Shapley Additive Explanation (SHAP) for interpretation. Time-to-onset, clinical priority, and exploratory transcriptomic signatures were also assessed.

**Results:**

In VAERS, 9446 AVA-related AE reports were identified, including 180 depression-related cases [total depression population *n* = 180, ROR 5.02 (95% *CI*: 4.33–5.81)]. Males (78.2%) and individuals aged 31–45 years (49.1%) were most represented, with a median time-to-onset of 10 days and 44.1% classified as serious. Stronger signals were observed in individuals aged 46–65 years [*n* = 35, ROR 10.31 (95% *CI*: 7.37–14.42)] and males [*n* = 142, ROR 6.11 (95%* CI*: 5.16–7.24)]. Machine learning highlighted serious AEs and age as key predictors. Transcriptomic analysis suggested immune-inflammatory pathway overlap, including neutrophil activation and *Yersinia* infection. In FAERS, levofloxacin [*n* = 634, ROR 1.37 (95% *CI*: 1.27–1.49)] and doxycycline [*n* = 332, ROR 1.53 (95% *CI*: 1.38–1.71)] showed positive depression-related reporting signals; “Depression suicidal” had moderate clinical priority.

**Conclusions:**

AVA was associated with a potential depression-related pharmacovigilance signal, particularly among males and adults, with early post-vaccination onset. These findings support targeted mental health monitoring after AVA, clearer safety communication in anthrax prevention programs, and vigilance when levofloxacin or doxycycline is used for anthrax therapy. As spontaneous reporting cannot establish causality or incidence, controlled epidemiological studies are needed.

**Graphical Abstract:**

**Figure:**
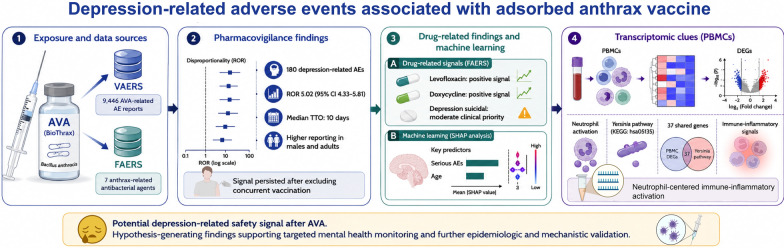

**Supplementary Information:**

The online version contains supplementary material available at 10.1186/s40249-026-01483-0.

## Background

Anthrax is a globally distributed zoonotic disease caused by *Bacillus anthracis* and is characterized by high transmissibility and potentially severe outcomes [1]. It remains endemic in resource-limited regions, where poor sanitation and frequent human-livestock contact increase transmission risks, making vaccination an important component of anthrax prevention in poverty-affected settings [2]. The 2001 United States bioterrorist attacks, in which *B. anthracis* spores contaminated more than 30,000 people and caused five deaths, further highlighted its potential threat as a bioweapon [3]. Clinically, anthrax can present as cutaneous, gastrointestinal, or pulmonary disease, with untreated pulmonary anthrax associated with particularly high mortality [4, 5].

Adsorbed anthrax vaccine (AVA, BioThrax) is licensed for preexposure prophylaxis in adults aged 18–65 years at high risk of *B. anthracis* exposure and is the only United States-authorized anthrax vaccine [6]. Although AVA has generally been considered to have an acceptable safety profile, post-marketing concerns regarding adverse events (AEs) remain clinically relevant [7, 8]. Local injection-site reactions and systemic symptoms such as fever, malaise, and myalgia have been commonly described, whereas mental health-related AEs have received less attention [9, 10]. Emerging clinical and preclinical evidence suggests that bacterial vaccination may be associated with depressive symptoms or depression-related AEs, as reported in the context of Bacillus Calmette-Guérin vaccination [11, 12]. However, evidence regarding depression-related AEs after AVA remains limited and inconsistent, with few studies specifically addressing depression as a post-vaccination safety outcome [13]. In resource-limited settings, where mental health services may be scarce, unrecognized depression-related AEs could affect vaccine confidence, adherence to vaccination schedules, and the implementation of anthrax prevention programs.

To address this evidence gap, we conducted an integrated real-world pharmacovigilance study to characterize depression-related AEs following AVA and to evaluate related safety signals for antibacterial agents used in anthrax treatment or chemoprophylaxis. We used the Vaccine Adverse Event Reporting System (VAERS) to identify AVA-associated depression-related AEs, and Food and the Drug Administration Adverse Event Reporting System (FAERS) to assess depression-related AEs associated with anthrax-related antibacterial agents [14–16]. Disproportionality and sensitivity analyses were used for signal detection, machine learning with Shapley Additive Explanation (SHAP) interpretation was applied to explore potential predictive features, and transcriptomic analysis was performed to identify exploratory immune-inflammatory clues. This integrated approach identified a potential depression-related safety signal following AVA, with distinct demographic and early time-to-onset patterns, and suggested additional depression-related reporting signals for levofloxacin and doxycycline, providing evidence to support post-vaccination mental health monitoring and risk communication in anthrax prevention programs.

## Methods

### Data source

This study used two large-scale post-marketing pharmacovigilance databases: the VAERS and the FAERS. The two databases were processed separately according to their respective data structures, while a consistent analytical framework was adopted for signal detection and downstream analyses. VAERS captures demographic information, vaccine exposure, and AE reports following vaccination [17, 18]. FAERS records drug-related AE reports across multiple linked tables, including demographic information, drug exposure, adverse reactions, outcomes, treatment timelines, reporter information, and indications [19, 20]. The study followed the REporting of A Disproportionality Analysis for DrUg Safety Signal Detection Using Individual Case Safety Reports in PharmacoVigilance (READUS-PV) guideline for disproportionality analysis based on individual case safety reports.

### Data extraction and processing

#### VAERS and FAERS data extraction and processing

AE reports were extracted from two post-marketing pharmacovigilance databases, VAERS and FAERS, using database-specific cleaning procedures while applying a consistent analytical framework for depression-related AE detection. For VAERS, AE reports spanning 1993–2025 were obtained from the VAERS database (https://vaers.hhs.gov/). The VAERSDATA, VAERSVAX, and VAERSSYMPTOMS tables were linked using VAERS_ID. Duplicate checking was performed at the report level according to the official VAERS data cleaning guidelines. Reports labelled as “Foreign” were excluded to restrict the analysis to United States-origin reports. Anthrax vaccine-related reports were identified by restricting the VAX_NAME field to “Anthrax”, followed by exclusion of entries marked “NO BRAND NAME” and vaccination-error reports without described AEs. Demographic and clinical information, including age, sex, vaccine dose, seriousness, and the interval from vaccination date to first symptom onset, was extracted when available. Depression-related AEs were identified using predefined Preferred Term (PT) keywords, and all remaining VAERS AE reports served as the reference group for disproportionality analysis.

For FAERS, AE reports were extracted for seven commonly used drugs for anthrax infection, including penicillin, levofloxacin, doxycycline, moxifloxacin, minocycline, ceftriaxone, and meropenem. Because these drugs are non-specific agents used for multiple indications and anthrax-related indication terms were rarely reported, no restriction was imposed on the indication field during FAERS screening. Duplicate reports in the FAERS DEMO table were removed using a deduplication procedure based on the case identifier (CASEID), FDA receipt date (FDA_DT), and primary report identifier (PRIMARYID): records sharing the same CASEID were sorted by FDA_DT, with the most recent report retained; when multiple records shared both the same CASEID and FDA_DT, the record with the highest PRIMARYID was retained [21, 22]. The DRUG, REAC, THER, RPSR, and OUTC tables were cleaned independently and linked using report identifiers for downstream drug-specific analysis. Depression-related AEs were identified using the same predefined PT keyword strategy, and only reports in which the selected drugs were listed as the primary suspect (PS) drug were included.

Detailed record counts and database-specific screening workflows for VAERS and FAERS are provided in Supplementary Methods and Supplementary Figs. 1–2.

#### Standardized AE coding

All AEs and associated signs or symptoms in both databases were coded using the Medical Dictionary for Regulatory Activities (MedDRA), version 27.0, maintained by the MedDRA Maintenance and Support Services Organization (MSSO; Herndon, VA, USA; https://www.meddra.org/). Each report could include one or more MedDRA preferred terms (PTs), which do not necessarily equate to medically confirmed diagnoses [23]. The MedDRA system’s hierarchical structure [encompassing System Organ Class (SOC), High-Level Group Term (HLGT), High-Level Term (HLT), and PT] was utilized for standardized AE classification [24], and all PTs were mapped to their corresponding SOCs and HLGTs for further exploration.

#### Disproportionality analysis

Descriptive statistical methods were used to characterize the features of depression-related AEs reports associated with the AVA (from VAERS) and the seven commonly used anthrax treatment drugs (from FAERS) [25]. Disproportionality analysis, which assesses the strength of association between specific medical products (vaccines or drugs) and target AEs, was conducted separately for VAERS and FAERS data via a four-cell table (presented in Supplementary Table 1). This analysis aimed to detect potential safety signals of depression linked to AVA vaccination and anthrax treatment drug use, respectively [26].

Four established disproportionality analysis methods—reporting odds ratio (ROR), proportional reporting ratio (PRR), Bayesian Confidence Propagation Neural Network (BCPNN), and Multinomial Gamma Poisson Shrinkage (MGPS)—were utilized to verify the potential association between AVA/drug exposure and depression-related AEs [27]. A depression-related AE was considered a potential safety signal if it met the positivity threshold defined by any of these methods, with larger values indicating a stronger signal and a more robust association. Supplementary Table 2 provides detailed information on the specific thresholds applied for each disproportionality analysis method. All analytical procedures were performed using R software (version 4.2.2; R Foundation for Statistical Computing, Vienna, Austria).

#### Clinical priority assessment of depression-related AEs induced by drugs for anthrax infection treatment

Given that anthrax vaccines are administered solely to individuals at high exposure risk or with confirmed infection, these populations consequently require follow-up clinical intervention. To close the translational gap between public health prevention and clinical treatment, we conducted a clinical priority evaluation of depression-AEs induced by seven anthrax treatment drugs. Four key indicators were measured to evaluate the clinical priority of positive signals: target event case numbers, the lower limit of ROR, the proportion of fatal outcome reports, and conformity to important medical event/designated medical event (IME/DME) criteria. AEs were assigned to low, moderate, or high priority levels according to cumulative scores of 0–2, 3–5, and 6–8, respectively [28] (Supplementary Table 3).

### Construction of the machine learning (ML) model

Our final dataset included records of individuals who received AVA, with separate modeling conducted to identify factors associated with depression-related AE reporting. The VAERS subset was randomly split into two partitions: 70% allocated for model training and validation and the remaining 30% reserved for independent testing, to reduce potential imbalance in data characteristics across partitions. Seven machine learning algorithms were implemented using EasyR software (version 2.0.1; EasyR development team, Wuhan, China) to predict depression-related AE reporting among AVA recipients [29]. These algorithms included least absolute shrinkage and selection operator (LASSO) logistic regression, k-nearest neighbors (KNN), support vector machine (SVM), random forest (RF), naive Bayes (NB), extreme gradient boosting (XGBoost), and decision tree. These seven algorithms were selected because they represent commonly used and complementary machine learning frameworks, including penalized regression, instance-based learning, kernel-based classification, ensemble tree-based learning, probabilistic classification, boosting, and interpretable decision-tree modeling. Their combined use allowed us to compare predictive performance across different modeling assumptions and to evaluate whether important features were consistently identified across algorithms, thereby improving the robustness of the exploratory analysis. Model performance was evaluated using sensitivity, specificity, positive predictive value (PPV), negative predictive value (NPV), precision, recall, F1 score, balanced accuracy, and overall accuracy. Hyperparameters were optimized via fivefold cross-validation and grid search, with five repeated train-test splits to assess model stability.

The SHAP method was used for interpretability: SHAP values quantify feature contributions to predictions, visualized in a summary plot where purple dots denote higher feature values and yellow dots lower ones [30].

### Variable preprocessing and survival analysis for depression onset risk assessment

Spearman's rank correlation analysis was first conducted on candidate variables to explore their inter-associations. Subsequently, LASSO regression was applied to these variables for selection and multicollinearity reduction. The variables filtered via this two-step process were then incorporated into survival analysis, aimed at quantifying the risk of depression onset over time. This analysis utilized the time-to-onset of depressive AEs following AVA vaccination or anthrax drug administration as the time scale and depression occurrence as the outcome event.

### Gene expression data analysis

#### GEO dataset screening and differential gene expression analysis

Gene expression data utilized in the present study were retrieved entirely from the Gene Expression Omnibus (GEO) database hosted by the National Center for Biotechnology Information (NCBI) of the United States [31]. Given that no GEO datasets directly linking anthrax vaccine exposure to depression were available, relevant datasets associated with anthrax infection and depression were screened based on consistency with the research topic and the transcriptional profiles of peripheral blood mononuclear cells. The AVA is essentially a subunit vaccine targeting *B. anthracis* protective antigen, which triggers immune and inflammatory responses similar to those induced by anthrax infection, thus supporting the use of infection-related datasets as a surrogate [32].

Differential gene expression analysis was performed on the normalized GSE34407 and GSE38206 datasets utilizing the "limma" package in R software (4.2.2) [33]. Comprehensive consideration of gene expression abundance, statistical reliability and biological relevance led to the determination of consistent screening criteria for both datasets: absolute value of log fold change no less than 1.5 and *P*-value less than 0.05. The resulting differentially expressed genes were visualized through volcano plots and heatmaps. Volcano plots illustrate the relationship between differential expression amplitude and statistical significance, while heatmaps depict the expression clustering characteristics of differentially expressed genes across different samples. Subsequently, upregulated and downregulated genes from the two datasets were subjected to intersection analysis separately to identify common co-expressed differential genes.

#### GO and KEGG enrichment analyses

To characterize the biological functions of the upregulated genes in *Bacillus anthracis* lethal toxin (LeTx)-treated peripheral blood mononuclear cells (PBMCs) and in PBMCs from patients with major depressive disorder (MDD), Gene Ontology (GO) functional annotation and Kyoto Encyclopedia of Genes and Genomes (KEGG) pathway enrichment analyses were performed separately for the two upregulated gene sets. The Database for Annotation, Visualization and Integrated Discovery (DAVID; version 2021 update; Laboratory of Human Retrovirology and Immunoinformatics, National Institute of Allergy and Infectious Diseases, National Institutes of Health, Frederick, MD, USA; https://davidbioinformatics.nih.gov/) was used for enrichment analysis [34]. Briefly, the upregulated gene lists were uploaded to the DAVID functional annotation module to identify significantly enriched GO terms and KEGG pathways. Enriched GO terms and KEGG pathways from the two gene sets were then compared to explore potential functional overlap between LeTx exposure and MDD-related transcriptomic alterations [35]. The resultant data were collated and visualized using R software (4.2.2).

## Results

### Temporal and geographic patterns of AVA-related AE reports

#### Product and manufacturer profile of AVA AE reports

**Fig. 1:**
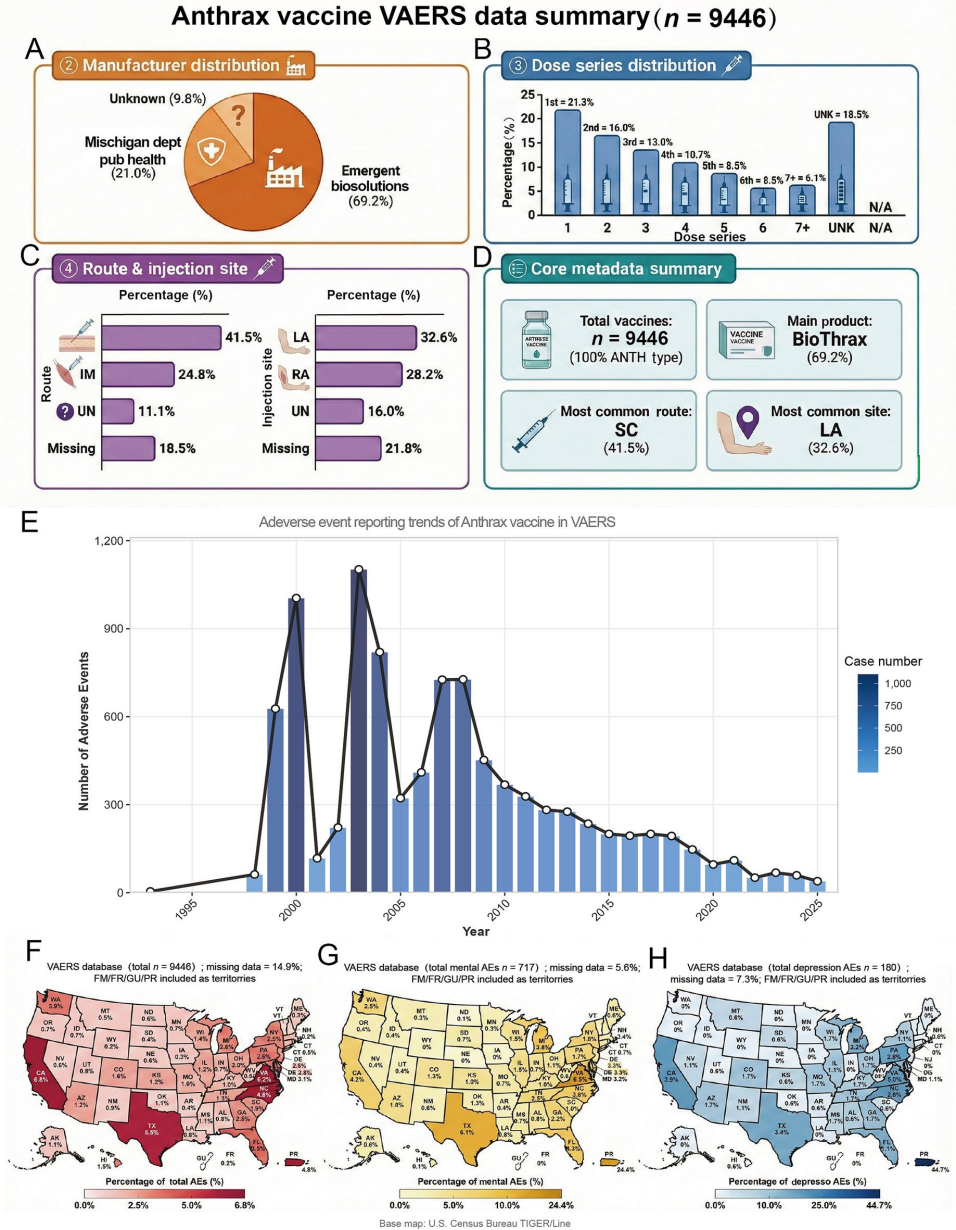
Summary characteristics of anthrax vaccine-associated adverse event reports in the VAERS database. **A** Pie chart showing the distribution of vaccine manufacturers. **B** Bar chart depicting the proportion of reports across vaccine dose series. **C** Stacked bars for the administration route. **D** Key metadata highlights. **E** Time-series plot of annual AVA AE report counts (1993–2025). **F** American state/territory maps showing the percentage of total AVA AEs. **G** American state/territory maps showing the percentage of mental health-related AEs. **H** American state/territory maps showing the percentage of depression-specific AEs. The base maps in **F**–**H** were generated using publicly available U.S. state and territory boundary data from the U.S. Census Bureau TIGER/Line Shapefiles and were adapted and visualized by the authors. *AE* adverse event, *AVA* adsorbed anthrax vaccine, *VAERS* Vaccine Adverse Event Reporting System

Spanning the period covered by the VAERS database from 1993 to 2025, our study extracted a total of 9446 AE reports linked to AVA vaccination. Figure 1 summarizes the AVA vaccine VAERS data characteristics: Emergent BioSolutions (69.2%) was the major manufacturer (Fig. 1A); the 1st dose (21.3%) and unknown dose series (18.5%) were the most common (Fig. 1B); subcutaneous injection (41.5%) was the primary administration route, and the left arm (32.6%) was the main vaccination site (Fig. 1C); meanwhile, BioThrax (69.2%) served as the main vaccine product, consistent with the manufacturer distribution (Fig. 1D). (Details in Supplementary Table 4).

The temporal trends of annual AVA AEs exhibit distinct fluctuations (Fig. 1E): a sharp surge occurred from 1998 to 2000, with the number of reports peaking at 1004; a second peak of 1102 reports was observed in 2003; a moderate elevation at 727 reports followed in 2008; thereafter, a sustained downward trend emerged, with fewer than 100 reports recorded annually in recent years.

Geographically, the distribution of AVA-related AEs across United States and territories varies by event type (Fig. 1F–H): For total AEs (Fig. 1F), Virginia (6.2%) and California (6.8%) accounted for the largest shares, while Texas (5.5%) and North Carolina/Puerto Rico (4.8% each) also contributed notably. For mental AEs (Fig. 1G), Puerto Rico had the highest proportion (24.4%), followed by Virginia (6.5%) and Texas (6.1%). For depression-specific AEs (Fig. 1H**)**, Puerto Rico again accounted for the largest share (44.7%), with Virginia (5.0%) and Texas (3.4%) being the most represented United States.

#### Age and sex distribution of AVA-associated depression AEs

After filtering AE types to include only depression-associated outcomes, the clinical features of 180 depression-related AEs linked to AVA vaccination are summarized in Table 1: males (78.2%, 141 cases) are the most affected gender, with females accounting for 17.3% (31 cases) and 4.5% (8 cases) of gender data unspecified; individuals aged 31–45 years dominate the age distribution (49.1%, 89 cases), followed by 18–30 years (33.5%, 60 cases), with no cases reported in patients younger than 18 or older than 65; the most common outcomes are disability (30.2%, 55 cases) and life-threatening events (17.9%, 32 cases), while 44.1% (79 cases) of these AEs are classified as serious.

**Table 1:** Clinical characteristics of depression-related adverse events in patients injected with anthrax vaccine from the VAERS database

Characteristics	Case number	Proportion (%)
Number of events	180	
Gender (%)		
Female	31	17.3
Male	141	78.2
Not specified	8	4.5
Age, years		
< 18	0	0
18–30	60	33.5
31–45	89	49.1
46–65	18	10.1
> 65	0	0
Not specified	13	7.3
Reported states		
Puerto Rico	81	4.4
Virginia	9	5.0
California	7	3.9
Texas	6	3.4
Carolina	5	2.8
Others	72	40.2
Serious AEs		
No	101	55.9
Yes	79	44.1
Single vaccine injection		
No	63	35.2
Yes	117	64.8
Outcome		
Death	1	0.6
Life threatening	32	17.9
Hospitalization	31	17.3
Prolonged hospitalization	3	1.7
Disability	55	30.2
Recovered	10	5.6
Not available	48	26.8

*AE* adverse event; *VAERS* Vaccine Adverse Event Reporting System

### AVA was associated with positive depression-related reporting signals

#### Depression and major depression showed positive PT-level signals

Based on clustering characteristics, we optimized the classification of Preferred Terms (PTs) under the depression category and identified 180 cases of depression-related AEs. Unless otherwise specified, the bracketed values indicate the 95% confidence interval for ROR, the *χ*^2^ statistic for PRR, the EBGM05 value for EBGM, and the IC025 value for IC. Overall, depression-related AEs showed a positive reporting signal in the total depression population [*n* = 180, ROR 5.02 (4.33–5.81), PRR 5.00 (573.98), EBGM 4.92 (4.35), IC 2.30 (2.08)]; among these, all depression-related PT subtypes with a case count of ≥ 3 are summarized in Table 2.

**Table 2:** Signal strengths of depression-related adverse events associated with anthrax vaccine ranked by the number of case reports at the PT level in the VAERS database

Preferred terms (PTs)	Case reports	ROR (95% *CI*)	PRR (*χ*^2^)	EBGM (EBGM05)	IC(IC025)
Depression related AEs (Unclassified type)	180	5.02 (4.33–5.81)	5 (573.98)	4.92 (4.35)	2.3 (2.08)
Depression*	169	6.96 (5.97–8.12)	6.94 (835.3)	6.77 (5.96)	2.76 (2.53)
Depressed mood	7	0.72 (0.34–1.51)	0.72 (0.76)	0.72 (0.39)	− 0.47 (-1.49)
Major depression*	4	4.55 (1.69–12.24)	4.55 (10.87)	4.48 (1.96)	2.16 (0.86)

Asterisks (*) indicate statistically significant signals. *AE* adverse event, *CI* confidence interval, *EBGM* empirical Bayesian geometric mean, *EBGM05* lower limit of the 95% confidence interval of EBGM, *IC* information component, *IC025* lower limit of the 95% confidence interval of IC, *PRR* proportional reporting ratio, *PT* Preferred Term, *ROR* reporting odds ratio, *VAERS* Vaccine Adverse Event Reporting System

**Fig. 2:**
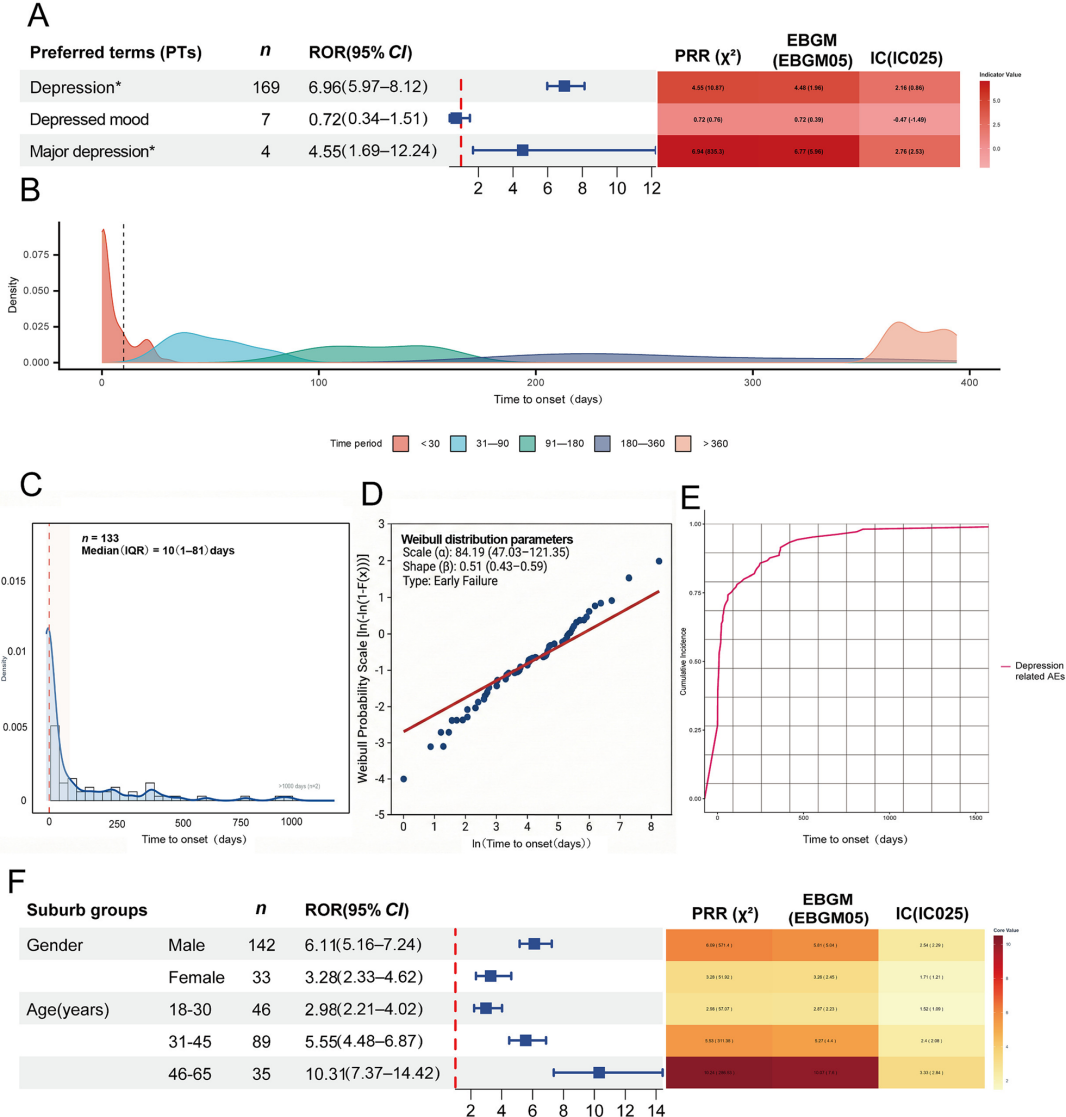
Signal characteristics and TTO patterns of AVA-associated depression-related adverse events. **A** Heatmap and forest plot of signal metrics (ROR, PRR, EBGM, IC) for three depression-related PTs. **B** Density plot of TTO. **C** Histogram of TTO. **D** Weibull model parameters. **E** Curve showing cumulative incidence of depression-related AEs. **F** Forest plot of signal metrics by gender. *AE* adverse event, *AVA* adsorbed anthrax vaccine, *EBGM* empirical Bayesian geometric mean,* IC* information component, *PRR* proportional reporting ratio, *PT* Preferred Term, *ROR* reporting odds ratio, *TTO* time to onset

To better illustrate the signal magnitudes of different depression subtypes, Fig. 2A displays the signal detection results of three categories of depression-related AEs associated with AVA vaccines. Among these, Depression [*n* = 169, ROR 6.96 (5.97–8.12), PRR 6.94 (835.30), EBGM 6.77 (5.96), IC 2.76 (2.53)] had the largest case volume with moderate signal strength. By contrast, Depressed Mood [*n* = 7, ROR 0.72 (0.34–1.51), PRR 0.72 (0.76), EBGM 0.72 (0.39), IC − 0.47 (− 1.49)] showed no statistically significant signal. Major Depression [*n* = 4, ROR 4.55 (1.69–12.24), PRR 4.55 (10.87), EBGM 4.48 (1.96), IC 2.16 (0.86)] presented a significant positive signal.

#### Depression-related AEs mainly occurred early after AVA vaccination

Beyond signal characteristics, the time to onset (TTO) of AVA-associated depression-related AEs also exhibited distinct patterns: a prominent peak in density occurred within the first 30 days post-vaccination, with a smaller secondary peak emerging after 360 days; most events arose within 180 days, and density remained low across later periods (Fig. 2B).

The median TTO was 10 days (interquartile range: 1–81 days) among 113 cases, with most events concentrated in the early post-vaccination period (Fig. 2C). The Weibull distribution analysis (Fig. 2D) yielded a shape parameter (β) of 0.51 (95% *CI*: 0.43–0.59) and a scale parameter (α) of 84.19 (95% *CI*: 47.03–121.35); the β value < 1 indicated an "early failure" pattern, reflecting a declining risk of AE occurrence over time. During the study observation period, the cumulative incidence of depression-related PT was quantified and visualized in Fig. 2E.

#### Positive signals persisted in subgroup and sensitivity analyses

As presented in Fig. 2F, the stratified analysis revealed distinct variations in signal strength across demographic subgroups of AVA-associated depression-related AEs. For the total population (*n* = 259), the signal metrics varied by subgroup: in gender stratification, males (*n* = 142) exhibited an ROR of 6.11 (95% *CI*: 5.16–7.24), while females (*n* = 33) had a lower ROR of 3.28 (95% *CI*: 2.33–4.62). In age stratification, the 46–65 years group (*n* = 35) showed the highest ROR of 10.31 (95% *CI*: 7.37–14.42), followed by the 31–45 years group (*n* = 89, ROR = 5.55, 95% *CI*: 4.48–6.87) and the 18–30 years group (*n* = 46, ROR = 2.98, 95% *CI*: 2.21–4.02). These results indicate that gender and age are key demographic factors contributing to heterogeneity in the association between single-dose AVA vaccination and depression-related AEs (Specific demographic information is provided in the Supplementary Table 5 to 9).

After excluding reports involving concurrent administration of other vaccines, our study identified 119 individual cases of depression-related AEs (Supplementary Table 10). Of note, even after accounting for the potential confounding impact of co-administered vaccines, the positive safety signals linking the AVA vaccine to depression remained statistically significant [*n* = 119, ROR 4.97 (4.14–5.96), PRR 4.95 (370.21), EBGM 4.9 (4.21), IC 2.29 (2.03)]. This finding suggests that the AVA-related reporting signal persisted after excluding reports with co-administered vaccines, although residual confounding cannot be fully excluded.

### Machine learning predictors of AVA-associated depression AEs

#### Predictive performance of random forest and XGBoost models

**Fig. 3:**
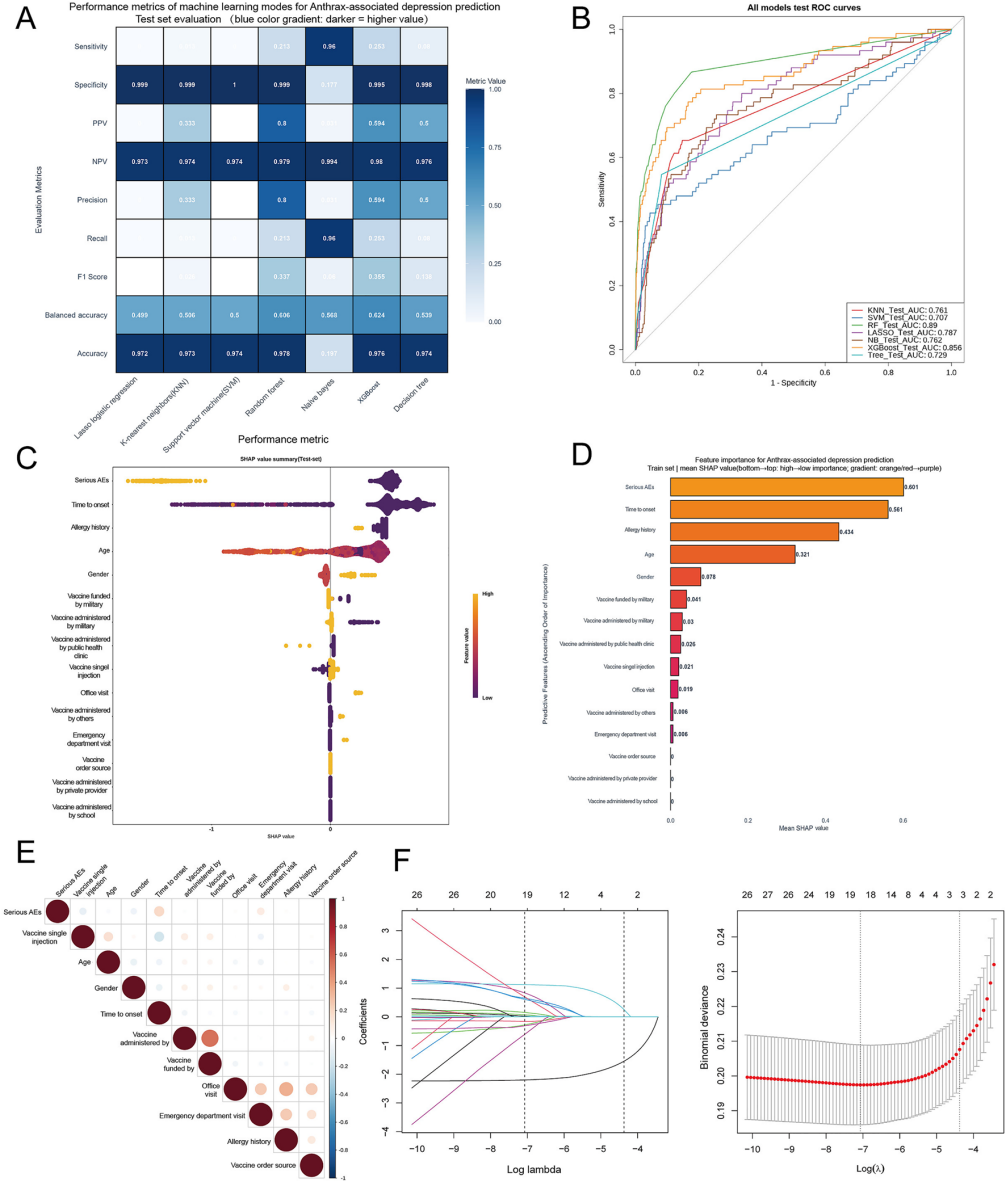

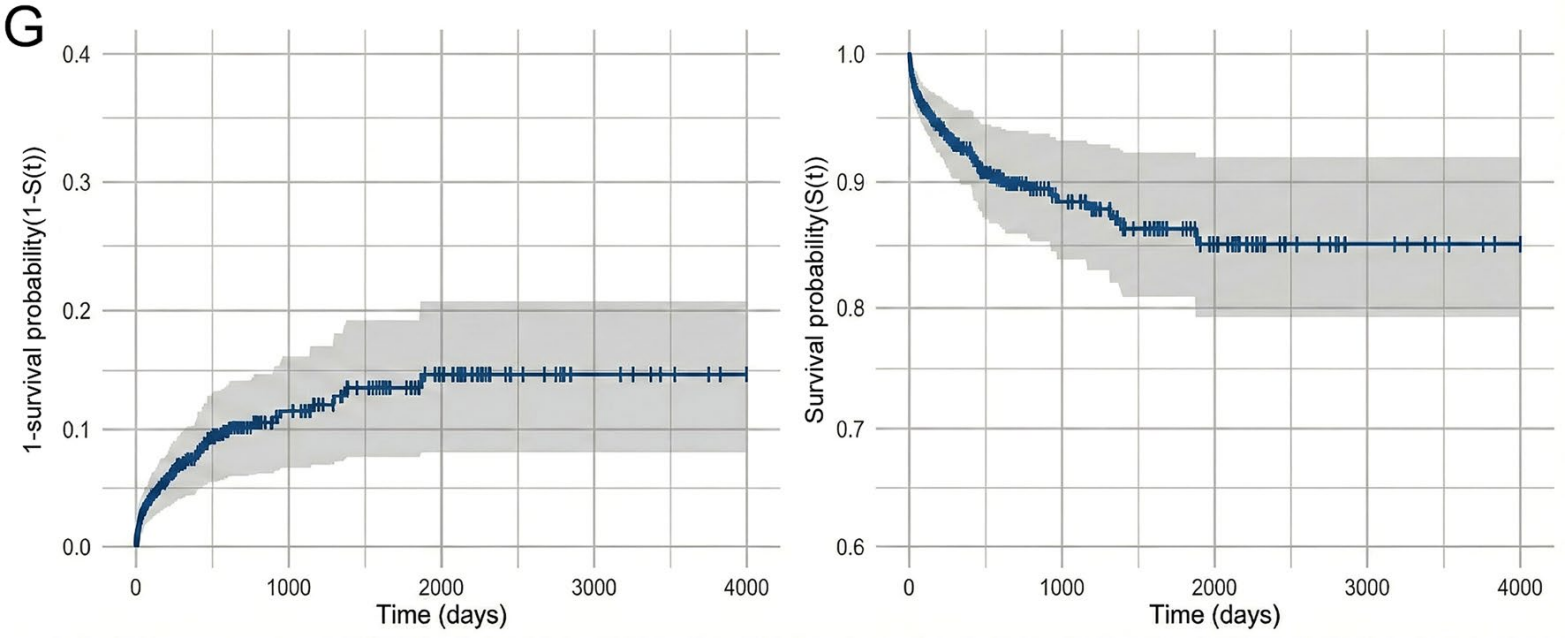
Machine learning model performance, feature interpretability, and survival analysis for AVA-associated depression-related adverse events. **A** Heatmap of test-set performance for seven ML algorithms. **B** Receiver operating characteristic curves for all ML models. **C** SHAP plot showing feature contributions to predictions. **D** Bar plot of mean SHAP values. (Panel E) Correlation matrix of predictive variables. **F** Regularization path (left) and binomial deviance (right) plots for LASSO predictor selection. **G** Left panel (1 − survival probability) shows rapid early cumulative incidence of depression-related AEs; right panel (survival probability S(t)) depicts declining “no AE” probability over time. *AE* adverse event, *AVA* adsorbed anthrax vaccine, *AUC* area under the curve, *LASSO* least absolute shrinkage and selection operator, *ML* machine learning, *ROC* receiver operating characteristic, *SHAP* Shapley Additive Explanation, *TTO* time to onset

Figure 3A presents the comparative performance metrics of seven ML models for predicting AVA-associated depression-related AEs, quantifying and visualizing indicators (including Sensitivity, Specificity, PPV, NPV, Precision, Recall, F1 Score, Balanced Accuracy, and Accuracy) in the test set.

Among the evaluated models, XGBoost exhibited relatively balanced comprehensive performance: it achieved a Balanced Accuracy of 0.624 (the highest among all models), paired with high Specificity (0.995) and Accuracy (0.976), though its Sensitivity (0.253) and F1 Score (0.355) remained moderate. Naive Bayes showed the highest Sensitivity (0.96) and Recall (0.96) but performed poorly in other metrics—including the lowest Specificity (0.177) and Accuracy (0.197)—leading to a low F1 Score (0.0596). Random Forest had moderate Sensitivity (0.213), coupled with high Specificity (0.999) and the highest PPV/Precision (0.8), though its F1 Score (0.3368) was limited by low Sensitivity (Supplementary Table 11).

LASSO Logistic Regression and SVM had poor predictive utility: LASSO showed 0 Sensitivity/Recall/PPV/Precision, while SVM lacked PPV/Precision values and had 0 Sensitivity/Recall. KNN and Decision Tree demonstrated mediocre performance across metrics: KNN had minimal Sensitivity (0.0133) and a low F1 Score (0.0256), while Decision Tree had low Sensitivity (0.08) and an F1 Score of 0.138, despite high Specificity (0.998) and Accuracy (0.974).

Figure 3B displays the test set receiver operating characteristic (ROC) curves of all ML models for predicting AVA-associated depression-related AEs, with the area under the curve (AUC) values labeled for each model. Among these, Random Forest (RF) achieved the highest test AUC (0.89), followed by XGBoost (0.856) and LASSO Logistic Regression (0.787), indicating strong discriminative ability for these two models. The remaining models showed moderate to lower AUCs: KNN (0.761), SVM (0.707), Tree (0.729), and Naive Bayes (NB, 0.762).

In combination with the performance metrics, Random Forest and XGBoost stand out as relatively optimal frameworks: Random Forest exhibits a high AUC (0.89) paired with strong Specificity and PPV, while XGBoost has a competitive AUC (0.856) alongside the highest Balanced Accuracy. Other models either show weaker discriminative power or significant trade-offs between metrics, limiting their practical utility for predicting AVA-associated depression-related AEs.

#### Serious AEs and time-to-onset were the most influential features

To clarify the contribution of each feature to the AVA-associated depression prediction model, we used SHAP-based visualization to assess feature importance in the model. The SHAP value swarm plot (Fig. 3C) and mean SHAP value ranking plot (Fig. 3D) both highlight the key features driving predictions: Serious AEs (mean SHAP = 0.601) and Time to Onset (mean SHAP = 0.561) emerged as the two most influential features, followed by Allergy History (mean SHAP = 0.434) and Age (mean SHAP = 0.321).

Notably, clinical indicators (Serious AEs, Time to Onset) and patient allergy history dominated the top of the importance ranking, while vaccine administration-related features had minimal impact (mean SHAP ≤ 0.078).

#### LASSO highlighted serious AEs as the key predictor

To clarify potential associations among features linked to AVA-associated depression-related AEs, a correlation heatmap of predictive variables was generated (Fig. 3E)—this visualization intuitively captures both the strength and direction of correlations between these features.

For pinpointing key predictors of these AEs, LASSO regression analysis was performed on candidate features (including Serious AEs, Time to Onset, and demographic variables). The regularization path plot (Fig. 3F, left) illustrates how feature coefficients shrink with increasing penalty (log λ), while the binomial deviance plot (Fig. 3F, right) identifies the optimal λ values via tenfold cross-validation.

The LASSO model retained 1 feature (Serious AEs) at λ_1se and 13 features at λ_min, providing a flexible framework to balance model simplicity and predictive performance for identifying AVA-associated depression-related AE risk factors.

#### Serious AEs and age were associated with earlier AE onset

Survival analysis was conducted using “TTO from AVA vaccination to the occurrence of depression-related AEs” as the time scale, with “depression-related AE onset” as the outcome event. The candidate predictors included in this analysis were the top 10 risk factors identified by LASSO regression, which were selected based on the largest λ values. Table 3 summarizes the Cox regression results, quantifying the hazard ratios (HRs) and statistical significance of candidate predictors: Serious AEs (HR = 3.829, 95% *CI*: 2.644–5.546, *P* < 0.0001) and Age (HR = 1.031, 95% *CI*: 1.013–1.048, *P* = 0.0004) were significantly associated with an increased risk of depression-related AEs. Other factors showed no statistically significant associations.

**Table 3:** Predictors of time to onset of depression-related adverse events: a survival analysis using Cox regression

Preferred terms (PTs)	Regression coefficient	Standard error of regression coefficient	z value	HR (exp(coef))	1/HR	95% *CI* (lower)	95% *CI* (upper)	*P*-value
Serious AEs	1.3427	0.1889	7.1062	3.8293	0.2611	2.6441	5.5456	< 0.0001
Vaccination by injection alone	− 0.1869	0.1844	− 1.0136	0.8295	1.2055	0.5779	1.1907	0.3108
Age(years)	0.0302	0.0086	3.5126	1.0307	0.9703	1.0134	1.0482	0.0004
Gender	0.0452	0.2190	0.2063	1.0462	0.9558	0.6811	1.6072	0.8365
Administered by others	− 0.3886	0.7373	− 0.5271	0.6780	1.4750	0.1598	2.8760	0.5981
Administered by public health clinic	0.3063	1.0400	0.2945	1.3583	0.7362	0.1769	10.4292	0.7684
Administered by private provider	− 13.7879	1602.9665	− 0.0086	0.0000	972,783.8202	0.0000	Inf	0.9931
Funded by others	− 0.1857	0.4327	− 0.4292	0.8305	1.2041	0.3557	1.9394	0.6678
Funded by public health clinic	0.3125	1.0620	0.2943	1.3669	0.7316	0.1705	10.9575	0.7685
Funded by private provider	− 0.2084	1.2244	− 0.1702	0.8119	1.2316	0.0737	8.9476	0.8649

AE: adverse event; CI: confidence interval; HR: hazard ratio; Inf: infinity; SER: standard error of regression coefficient. HR was calculated as exp(regression coefficient), where HR > 1 indicates an increased risk of depression-related adverse events and HR < 1 indicates a decreased risk. Inf represents infinity due to extreme HR estimation

Figure 3G visually illustrates the time-event pattern: the left panel (1—survival probability) shows the cumulative incidence of depression-related AEs rising rapidly in the early post-vaccination period before stabilizing; the right panel (survival probability S(t)) depicts the corresponding decline in the probability of “no AE onset” over time.

### Depression-related signals for anthrax-related antibacterial agents

#### Positive drug-related signals for depression-related PTs

Using the FAERS database, we evaluated depression-related AE reports associated with seven commonly used anthrax-related antibacterial agents, including penicillin, levofloxacin, doxycycline, moxifloxacin, minocycline, ceftriaxone, and meropenem. A total of 1763 depression-related AE reports were identified for these seven agents, including eight depression-related PT subtypes with a case count of ≥ 3. (Clinical characteristics in Supplementary Table 15).

**Fig. 4:**
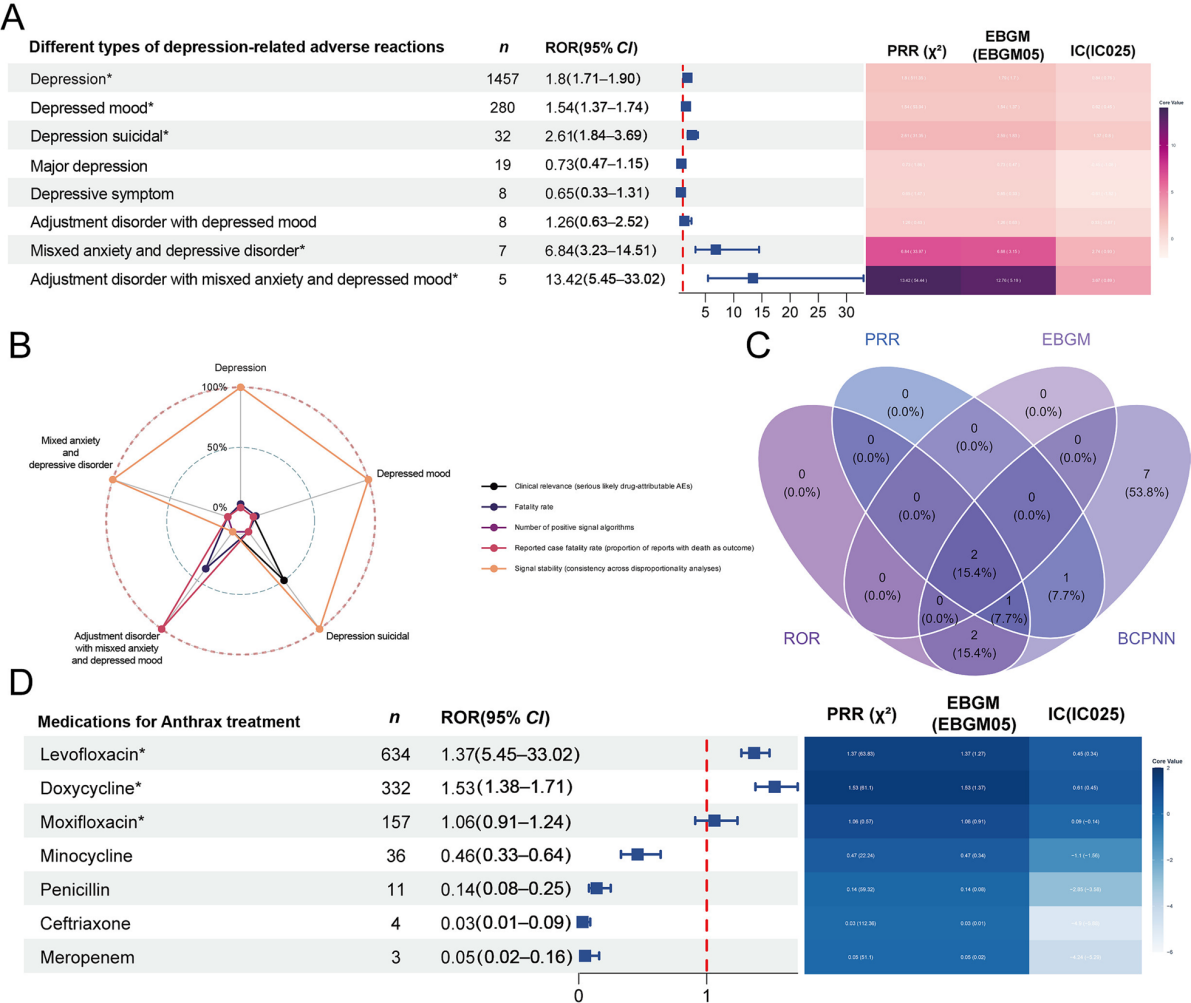
Pharmacovigilance analysis of depression-related adverse events associated with anthrax treatment drugs in the FAERS database. **A** Heatmap and forest plot of signal metrics for eight depression-related PTs. **B** Radar plot evaluating four priority indicators. **C** Overlap of positive signals across four disproportionality methods. **D** Forest plot and heatmap of signal metrics for seven anthrax treatment drugs. *AE* adverse event, *EBGM* empirical Bayesian geometric mean, *FAERS* Food and Drug Administration Adverse Event Reporting System, *IC* information component, *PRR* proportional reporting ratio, *PT* Preferred Term, *ROR* reporting odds ratio

Among these 8 depression subtypes, Depression [*n* = 1457, ROR 1.8 (1.71–1.9), PRR 1.8 (511.35), EBGM 1.79 (1.7), IC 0.84 (0.76)] had the largest case volume and a moderate positive signal. Depressed Mood [*n* = 280, ROR 1.54 (1.37–1.74), PRR 1.54 (53.04), EBGM 1.54 (1.37), IC 0.62 (0.45)] showed a mild positive signal with the second-highest case count. Adjustment Disorder with Mixed Anxiety and Depressed Mood [*n* = 5, ROR 13.42 (5.45–33.02), PRR 13.42 (54.44), EBGM 12.76 (5.19), IC 3.67 (0.89)] and Mixed Anxiety and Depressive Disorder [*n* = 7, ROR 6.84 (3.23–14.51), PRR 6.84 (33.97), EBGM 6.68 (3.15), IC 2.74 (0.93)] exhibited the strongest positive signals despite relatively small case volumes. Depression Suicidal [*n* = 32, ROR 2.61 (1.84–3.69), PRR 2.61 (31.35), EBGM 2.59 (1.83), IC 1.37 (0.8)] also presented a distinct positive signal (Fig. 4A). By contrast, Major Depression, Depressive Symptom, and Adjustment Disorder with Depressed Mood showed no statistically significant signals.

#### Depression suicidal had the highest clinical priority

Given the important reference value of these drugs for clinical medication decision-making in the subsequent treatment of anthrax infection, this study further conducted a clinical priority assessment of their associated signal results. We assessed the clinical priority of 5 depression-related AE subtypes with positive signals, with results summarized in Table 4 and visually illustrated in the accompanying radar chart. Among these subtypes, only Depression Suicidal was categorized as a moderate clinical priority; the remaining 4 subtypes were rated as low clinical priority. Specifically, Depression Suicidal achieved a moderate priority rating due to its strong signal stability (consistency across 2 disproportionality analysis methods) and designation as a serious likely drug-attributable AE (clinical relevance score = 1), key strengths clearly reflected in the radar chart, where it showed the highest performance in the "Signal stability" and "Clinical relevance" dimensions (Fig. 4B). To further characterize the reliability of these positive signals, Fig. 4C illustrates the overlap of positive signals across four disproportionality algorithms for depression-related AEs. Among the evaluated AEs, 2 subtypes yielded positive signals in all four algorithms.

**Table 4:** Priority ranking of adverse events based on disproportionality analysis and clinical relevance criteria

Types of depression	Number of positive signal algorithms	Signal stability (consistency across disproportionality analyses)	Fatality rate	Reported case fatality rate (proportion of reports with death as outcome)		Clinical relevance (serious likely drug-attributable AEs)	Total score	Priority
Depression	0	1	0.029	0		0	1	Low
Depressed mood	0	1	0.021		0	0	1	Low
Depression suicidal	0	2	0		0	1	3	Medium
Adjustment disorder with mix anxiety and depressed mood	0	0	0.38		1	0	1	Low
Mixed anxiety and depressive disorder	0	2	0		0	0	2	Low

*AE* adverse event

#### Levofloxacin and doxycycline showed positive drug-specific signals

Figure 4D summarizes the signal strengths of depression-related AEs associated with seven anthrax treatment medications, ranked by the number of case reports, using data from the FAERS database. Among the evaluated drugs, levofloxacin [*n* = 634, ROR 1.37 (1.27–1.49), PRR 1.37 (63.83), EBGM 1.37 (1.27), IC 0.45 (0.34)] and doxycycline [*n* = 332, ROR 1.53 (1.38–1.71), PRR 1.53 (61.1), EBGM 1.53 (1.37), IC 0.61 (0.45)] exhibited statistically significant positive signals. In contrast, moxifloxacin, minocycline, penicillin, ceftriaxone, and meropenem all presented significant negative signals, indicating a lower-than-expected reporting rate of depression-related AEs for these medications. (Clinical characteristics in Supplementary Table 16 to 22).

### Shared immune-related transcriptomic features

#### Overlapping upregulated genes

**Fig. 5:**
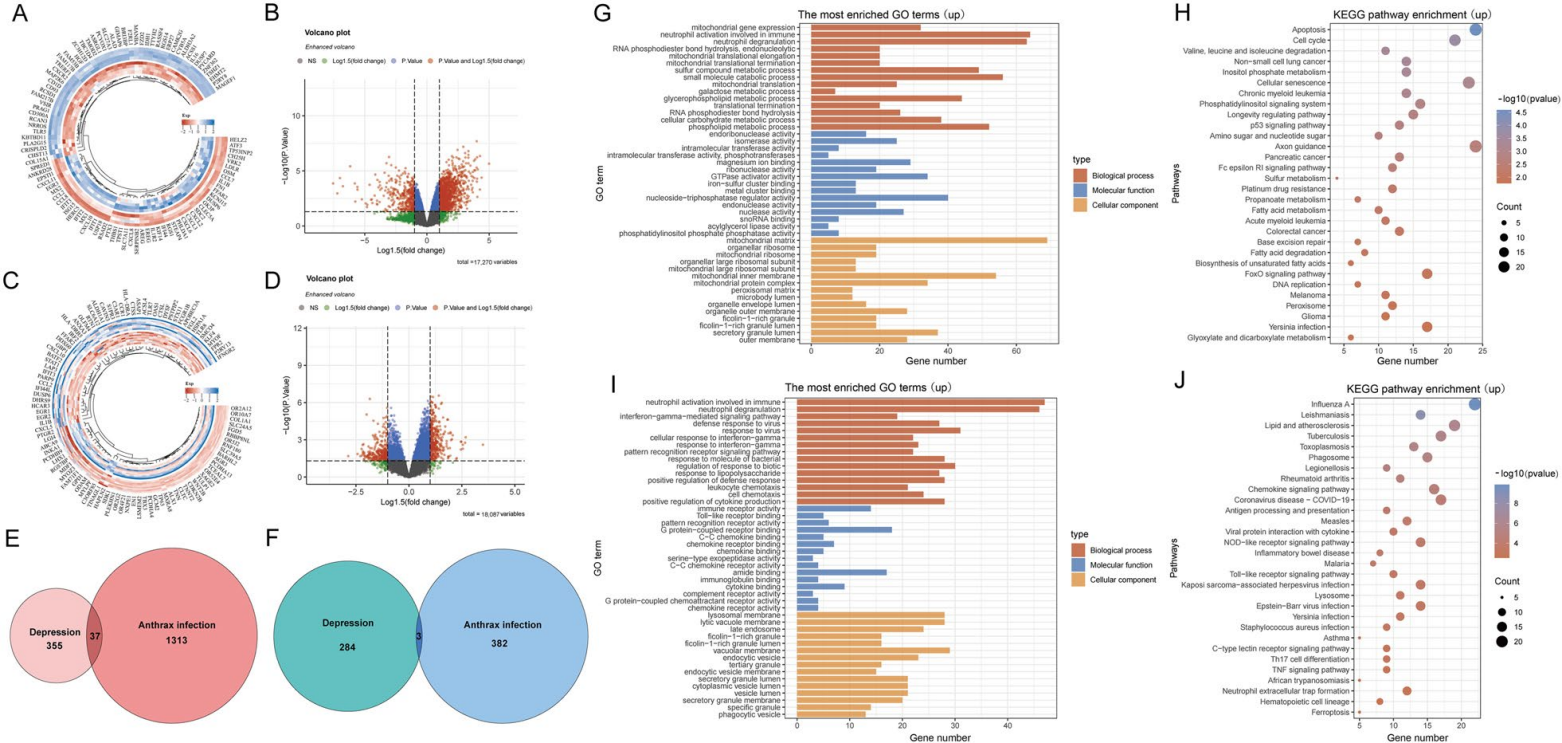
Transcriptomic profiling and convergent functional enrichment of *Bacillus anthracis* LeTx-treated PBMCs and MDD patient PBMCs. **A**, **B** (LeTx-treated PBMCs): **A** Heatmap of DGE in LeTx-treated vs. control PBMCs (GSE34407); **B** volcano plot identifying 1,313 upregulated and 382 downregulated genes. **C**, **D** (MDD patient PBMCs): **C** Heatmap of DGE in first-week MDD vs. healthy control PBMCs (GSE38206); **D** volcano plot identifying 355 upregulated and 284 downregulated genes. **E**, **F** (DEG intersection): Venn diagrams showing 37 overlapping upregulated genes (**E**) and 3 overlapping downregulated genes (**F**) between LeTx-treated and MDD PBMCs. **G**, **H** (LeTx-treated PBMC enrichment): **G** Top GO terms; **H** KEGG pathway enrichment. **I**, **J** (MDD PBMC enrichment): **I** Top GO term; **J** KEGG pathway enrichment. *DEG* differentially expressed gene, *DGE* differential gene expression,* GO* Gene Ontology,* KEGG* Kyoto Encyclopedia of Genes and Genomes, *LeTx* lethal toxin, *MDD* major depressive disorder, *PBMC* peripheral blood mononuclear cell

Two public transcriptomic datasets from the GEO database were re-analyzed to investigate gene expression profiles in distinct biological contexts. First, the GSE34407 dataset was utilized to explore the global transcriptional responses induced by *B. anthracis* lethal toxin (LeTx) in human peripheral blood mononuclear cells (PBMCs). Specifically, PBMCs were treated with 500 ng/ml LeTx for 4 h, with untreated cells serving as controls; a heatmap was generated to visualize the differential gene expression patterns across samples (Fig. 5A). Using a cutoff criterion of |log2 fold change (FC)|> 1.5 and adjusted *P*-value (*P*.adj) < 0.05, a total of 1313 genes were significantly upregulated and 382 genes were downregulated in LeTx-treated PBMCs compared to controls (Fig. 5B). Second, the GSE38206 dataset, previously shown to have good diagnostic performance for major depressive disorder (MDD), was re-analyzed to examine transcriptional alterations in PBMCs from patients with first-week MDD relative to age-matched healthy controls (first-week samples). A heatmap was constructed to illustrate the gene expression differences between groups (Fig. 5C), and applying the same cutoff threshold (|log2FC|> 1.5, *P*.adj < 0.05), 355 genes were identified as upregulated and 284 genes as downregulated in MDE patients’ PBMCs compared to controls (Fig. 5D). Notably, intersection analysis revealed 37 overlapping genes between the upregulated gene sets of MDD patients and LeTx-treated PBMCs (Fig. 5E), while only 3 common genes were observed in the downregulated gene sets of both groups (Fig. 5F). Supplementary Material 2 and Supplementary Material 3 offer comprehensive details on these significantly differentially expressed genes.

#### Neutrophil-related enrichment of shared upregulated genes

Notably, the two gene sets exhibited consistent enrichment patterns in both GO terms and KEGG pathways, providing potential evidence of convergent molecular mechanisms. For GO annotation, both gene sets were significantly enriched in the same biological process (BP) terms, including neutrophil activation involved in immune response and neutrophil degranulation (Fig. 5G for LeTx-treated PBMCs; Fig. 5I for MDD patient’ PBMCs), highlighting shared core immune regulatory processes. In the cellular component (CC) category, the two gene sets also shared identical enriched terms, namely ficolin-1-rich granule, ficolin-1-rich granule lumen, and secretory granule lumen (Fig. 3G, I), which are associated with neutrophil granule structure and function. In KEGG pathway enrichment analysis, the *Yersinia* infection pathway appeared among enriched pathways in both upregulated gene sets. This pathway was nominally enriched in the LeTx-treated PBMC upregulated gene set (GeneRatio = 17/574, *P* = 0.0166, FDR = 0.1788) and remained significant after FDR correction in the MDD PBMC upregulated gene set (GeneRatio = 11/197, *P* = 4.91 × 10⁻^4^, FDR = 0.00605) (Fig. 5H for LeTx-treated PBMCs; Fig. 5J for MDD patients’ PBMCs; detailed statistics are provided in Supplementary Material 4). This shared pathway, together with the consistent immune-related GO annotations, indicates that LeTx-induced transcriptional alterations and MDD-associated transcriptional dysregulation converge on immune response pathways centered on neutrophil activation and granule function, as well as *Yersinia* infection-related signaling, which may serve as the molecular bridge linking *B. anthracis* exposure and depressive-like transcriptional characteristics. All significantly enriched entries meeting the threshold of *P*.adj < 0.05.

## Discussion

This pharmacovigilance study comprehensively characterized depression-related AEs associated with AVA and integrate cross-database safety signals spanning prevention and clinical treatment via the United States VAERS and FAERS databases. The study leveraged two large-scale spontaneous reporting systems and incorporated machine learning (ML)-driven risk factor identification, survival analysis, and exploratory transcriptomic pathway analysis. Our study systematically analyzed 9446 AVA-related AE reports from VAERS, identifying 180 depression-specific cases, and further explored depression-related AEs from 7 common anthrax treatment drugs using FAERS. The FAERS analysis of anthrax-related antibacterial agents complements the VAERS findings by extending the safety evaluation from preventive vaccination to subsequent clinical treatment contexts. We identified disproportionate reporting signals of depression-related AEs following AVA (ROR = 5.02, 95% *CI*: 4.33–5.81), with distinct demographic reporting patterns, including higher reporting proportions among males and individuals aged 31–45 years, an early-onset temporal pattern, and signal persistence after excluding reports with concurrent vaccinations. Subgroup analyses suggested that sex and age may contribute to heterogeneity in AVA-associated depression-related AE reports, although these findings should be interpreted cautiously given the inherent limitations of spontaneous reporting databases. ML models highlighted serious AEs and age as key predictive factors. In addition, SHAP analysis indicated that serious AEs and time to onset, rather than vaccine administration-related variables, were the main contributors to model prediction, suggesting that clinical severity and temporal characteristics may be more informative for identifying AVA-associated depression-related AE reports. Survival analysis further supported the temporal relevance of serious AEs and age in relation to the onset of AVA-associated depression-related AEs. Transcriptomic analysis of GEO datasets further revealed convergent molecular pathways—neutrophil activation-related GO terms and the *Yersinia* infection KEGG pathway—between *B. anthracis* LeTx-treated PBMCs and PBMCs from patients with MDD.

### Contextualizing prior research on AVA and mental health

Previous studies on depression-related AEs induced by AVA vaccination are scarce and outdated, with only the CDC-initiated Anthrax Vaccination Program (AVP) launched in 2002 providing relevant evidence, yet its findings remain controversial [36]. Both prior CDC studies focused on mental health via the SF-36v2 survey but differed in design and emphasis: the observational AVP (*n* = 576) compared 437 AVA recipients to 139 unvaccinated Laboratory Response Network (LRN) workers, reporting a marginal unadjusted decline in mental scores at 30 months (vaccinated: − 2.11 vs. controls: − 0.24, *P* = 0.06) that became non-significant after confounder adjustment (*P* = 0.37) [37]; the randomized placebo-controlled AVA Human Clinical Trial (*n* = 1562) enrolled a broader healthy cohort, assigned to seven groups (full/reduced AVA or placebo), and found no significant differences in mental score changes across groups over 42 months (*P* = 0.64). Despite shared reliance on general mental function metrics and non-conclusive findings, the observational AVP had baseline mental score disparities (vaccinated: 55.0 vs. controls: 51.4, *P* < 0.0001) and limited generalizability to non-LRN populations, while the clinical trial’s rigorous design strengthened its null result credibility. However, although the SF-36v2 incorporates mood-related dimensions, it lacks targeted analysis of depressive symptoms and insights into molecular mechanisms [38]. In this context, our study focused specifically on depression-related AEs and integrated pharmacovigilance, machine learning, and transcriptomic analyses to provide additional real-world and exploratory mechanistic evidence.

### Serious AEs: a pivotal risk factor for AVA-associated depressive outcomes

The identification of serious AEs as the most influential feature in both SHAP analysis and LASSO regression suggests an important association between severe adverse reactions following AVA and subsequent depression-related AE reporting. This finding is consistent with the broader view that severe physiological perturbations after vaccination may contribute to neuroimmune disruption and mood-related symptoms [39]. Systemic inflammation, tissue injury, or multi-organ involvement may act as potent biological stressors, and disruption of neuroimmune homeostasis has been implicated in the pathogenesis of depression [40–42]. Unlike mild or local AEs, Serious AEs may trigger sustained pro-inflammatory responses, with cytokines and other immune mediators crossing the blood-brain barrier to alter neurotransmitter function and neuroplasticity, thereby increasing vulnerability to depressive symptoms [43, 44]. Additionally, experiencing a severe AE can induce significant psychological distress [45]. This distress may stem from fear of long-term health consequences, functional impairment, or healthcare-related burden, and can independently contribute to the onset of depression [46]. Together, these physiological and psychological stressors may jointly increase susceptibility to depression-related AEs [47].

### Exploratory transcriptomic clues related to depression-related AE signals

Because AVA targets the protective antigen of *B*. *anthracis* and elicits immune responses related to anthrax-associated immune activation, we utilized LeTx-treated PBMCs as a surrogate for AVA-induced transcriptional alterations. Notably, we identified 37 overlapping upregulated genes with functional enrichment analyses revealing consistent convergence on two core biological processes: neutrophil activation-related immune responses and *Yersinia* infection-associated signaling. For immune pathways, both datasets were significantly enriched in GO terms including “neutrophil activation involved in immune response” and “neutrophil degranulation”, as well as “ficolin-1-rich granule” and “secretory granule lumen”—key features of neutrophil-mediated inflammation [48, 49].

This aligns with the growing recognition that aberrant neutrophil activation and dysregulated granule release contribute to neuroinflammation, a central mechanism in depression pathogenesis [50]. Neutrophils activated by AVA-related immune stimulation may secrete pro-inflammatory cytokines and granule-derived factors capable of influencing blood-brain barrier permeability and central inflammatory responses [51, 52]. This peripheral immune infiltration triggers neuroinflammatory responses that disrupt synaptic integrity, hinder neuroplastic remodeling, and perturb the delicate balance of neurotransmitter systems—collectively shifting the central nervous system toward a state permissive for depressive symptom emergence [53]. IL-6 and TNF-α stand out as key cytokine mediators of this cascade, while ficolin-1, a major granule component, amplifies neuroinflammatory signaling [54, 55]; concurrent dysregulation of serotonin and dopamine pathways further compounds mood-related impairments by disrupting reward processing and emotional regulation [56, 57].

Additionally, the shared enrichment of the Yersinia infection KEGG pathway may reflect overlapping inflammatory signaling cascades linking anthrax-related immune activation and depression-related transcriptional changes. While *Yersinia* and *B. anthracis* are distinct pathogens, their infection-related signaling pathways overlap in activating NF-κB and MAPK cascades—key regulators of pro-inflammatory gene expression [58, 59]. AVA-induced immune activation may recapitulate elements of this pathway, leading to sustained inflammatory responses that parallel the neuroinflammatory state observed in depression [60, 61]. This convergent signaling provides a molecular bridge connecting vaccine-induced immune perturbation to depressive transcriptional phenotypes [62], complementing our prior finding that systemic inflammation via Serious AEs is a top risk factor for AVA-associated depression.

### Depression-related reporting signals for anthrax-related antibacterial agents

To characterize depression-related reporting signals across the continuum of anthrax prevention and clinical management, we extended our pharmacovigilance analysis to seven antibacterial agents used for anthrax treatment or chemoprophylaxis. This approach helps fill the translational gap between vaccine-related preventive safety signals and treatment-associated safety concerns [63].

Notably, among the evaluated medications, only levofloxacin and doxycycline exhibited statistically significant positive signals for depression-related AEs. This drug-specific discrepancy stems from the unique pharmacodynamic and pharmacokinetic properties of fluoroquinolones and tetracyclines, which mediate central nervous system (CNS)-related mood disturbances through multi-layered mechanisms that intersect with depression pathogenesis [64]. For levofloxacin, its lipophilic structure enables robust penetration of the blood-brain barrier, where it binds to and inhibits GABA receptors [65]. This inhibition reduces GABAergic neurotransmission, disrupting the balance between excitatory and inhibitory signaling in brain regions critical for mood regulation [66].

For doxycycline, while it also crosses the blood-brain barrier, its contribution to depression-related AEs is mediated primarily through dysregulation of the gut-brain axis and immune-neuronal crosstalk [67]. As a broad-spectrum antibiotic, doxycycline alters the gut microbiota composition by reducing beneficial commensal bacteria that produce neuroactive metabolites [68]. This microbial dysbiosis decreases peripheral tryptophan availability, and elevates levels of indoleamine 2,3-dioxygenase (IDO), an enzyme that diverts tryptophan toward kynurenine pathway metabolism [69]. The resulting reduction in central serotonin synthesis, combined with increased production of neurotoxic kynurenine metabolites, impairs synaptic plasticity and neurotransmitter balance, promoting depressive phenotypes [70].

The clinical priority assessment of depression-related AEs from anthrax treatment drugs highlights that only “Depression Suicidal” merits moderate priority, driven by its serious clinical impact and consistent signal stability—underscoring the need for proactive mental health monitoring in patients treated with levofloxacin or doxycycline. For other depression subtypes classified as low priority, clinicians can balance the urgency of anthrax treatment with routine mood surveillance, while prioritizing drugs like meropenem or ceftriaxone for high-risk groups to mitigate cumulative risk.

### Limitations of the study

While this study provides real-world evidence on depression-related AEs associated with AVA vaccination and anthrax-related therapeutic agents, several limitations should be acknowledged. Both VAERS and FAERS are spontaneous reporting systems and are therefore subject to inherent limitations, including underreporting, stimulated reporting, reporting bias, missing information, and variable report quality. Mild or delayed depression-related symptoms may be underreported, whereas events occurring after heightened public or clinical awareness may be overreported. Therefore, the number of reports in these databases should not be interpreted as the true incidence of depression-related AEs.

In addition, neither VAERS nor FAERS provides a true denominator of exposed individuals. Consequently, the present analyses cannot estimate absolute risk, incidence rates, or population-level risk differences. Disproportionality methods, including ROR, PRR, BCPNN, and MGPS, are designed to detect potential safety signals but cannot establish causality [71]. Accordingly, the observed signals should be interpreted as hypothesis-generating pharmacovigilance findings rather than evidence of a confirmed causal association between AVA or anthrax-related antibacterial agents and depression-related AEs.

Residual confounding also cannot be excluded. Important clinical and contextual factors, such as pre-existing mood disorders, baseline psychiatric symptoms, deployment-related stress, occupational exposure, concurrent medications, comorbidities, and concomitant vaccination, may influence both the occurrence of depression-related symptoms and the likelihood of reporting. This limitation is particularly relevant because AVA is frequently administered to military or occupationally exposed populations, in whom post-traumatic stress, operational stress, deployment-related experiences, and sleep disruption may influence both depression-related symptoms and AE reporting. Because these variables are incompletely captured in spontaneous reporting databases, they could not be fully adjusted for in the present study [72]. Finally, the available reports lack detailed clinical information on psychiatric history, symptom severity, diagnostic confirmation, treatment course, and long-term outcomes. Therefore, it was not possible to reliably distinguish new-onset depression from exacerbation of pre-existing depressive symptoms [73]. Moreover, because no public dataset directly links AVA exposure to depression-related outcomes, LeTx-treated PBMCs were used only as a surrogate model of anthrax-related immune perturbation. Thus, the transcriptomic analysis should be interpreted as exploratory and hypothesis-generating, providing mechanistic clues rather than definitive biological evidence.

## Conclusions

In the VAERS analysis, 9446 AVA-related AE reports were identified, including 180 depression-related AEs, with early post-vaccination onset and stronger reporting patterns among males and adults. Machine learning analyses highlighted serious AEs and age as key predictive features, while exploratory transcriptomic analyses suggested immune-inflammatory signatures involving neutrophil-related pathways. This study suggests a potential pharmacovigilance safety signal of depression-related AEs following AVA, with distinct demographic and early-onset reporting patterns. Complementary FAERS analysis suggested positive reporting signals for levofloxacin and doxycycline among antibacterial agents used for anthrax treatment or chemoprophylaxis, with “Depression Suicidal” rated as a moderate clinical priority. These findings should be interpreted as hypothesis-generating and warrant further validation in well-designed epidemiological and mechanistic studies.

## Supplementary Information

Supplementary material 1.

Supplementary material 2.

Supplementary material 3.

Supplementary material 4.

## Data Availability

Publicly available datasets were analyzed in this study. This data can be found here: The data utilized in these analyses are publicly accessible through the VAERS, FAERS, and GEO dataset, available at https://vaers.hhs.gov/, https://www.fda.gov/drugs/drug-approvals-and-databases/fda-adverse-event-reporting-system-faers-database, and https://www.ncbi.nlm.nih.gov/geo/.

## Abbreviations

AVA: Adsorbed anthrax vaccine
AE: Adverse event
AUC: Area under the curve
BCPNN: Bayesian Confidence Propagation Neural Network
CASEID: Case identifier
*CI*: Confidence interval
CNS: Central nervous system
DAVID: Database for Annotation, Visualization and Integrated Discovery
READUS-PV: REporting of A Disproportionality Analysis for DrUg Safety Signal Detection Using Individual Case Safety Reports in PharmacoVigilance
DEG: Differentially expressed gene
DGE: Differential gene expression
DME: Designated medical event
EBGM: Empirical Bayesian geometric mean
FAERS: Food and Drug Administration Adverse Event Reporting System
FC: Fold change
FDA_DT: FDA receipt date
GEO: Gene Expression Omnibus
GO: Gene Ontology
HLGT: High-Level Group Term
HLT: High-Level Term
HR: Hazard ratio
IC: Information component
IME: Important medical event
IQR: Interquartile range
KEGG: Kyoto Encyclopedia of Genes and Genomes
KNN: K-nearest neighbors
LASSO: Least absolute shrinkage and selection operator
LeTx: Lethal toxin
MGPS: Multinomial Gamma Poisson Shrinkage
MedDRA: Medical Dictionary for Regulatory Activities
ML: Machine learning
NB: Naive Bayes
NPV: Negative predictive value
PBMCs: Peripheral blood mononuclear cells
PPV: Positive predictive value
PRIMARYID: Primary report identifier
PRR: Proportional reporting ratio
PS: Primary suspect
PT: Preferred Term
RF: Random forest
ROC: Receiver operating characteristic
ROR: Reporting odds ratio
SHAP: Shapley Additive Explanation
SOC: System Organ Class
SVM: Support vector machine
TTO: Time to onset
VAERS: Vaccine Adverse Event Reporting System
XGBoost: Extreme gradient boosting

## References

[1] Fasanella A, Galante D, Garofolo G, Jones MH. Anthrax undervalued zoonosis. Vet Microbiol. 2010;140(3–4):318–31. 19747785 10.1016/j.vetmic.2009.08.016

[2] Senyange S, Nsubuga EJ, Kwesiga B, Bulage L, Ario AR. Knowledge, attitudes, and practices regarding anthrax among affected communities, Kazo district, South-Western uganda, May 2022. BMC Public Health. 2025;25(1):2249. 40604622 10.1186/s12889-025-23436-2 PMC12220345

[3] Inglesby TV, O’Toole T, Henderson DA, Bartlett JG, Ascher MS, Eitzen E, et al. Anthrax as a biological weapon, 2002: updated recommendations for management. JAMA. 2002;287(17):2236–52. 11980524 10.1001/jama.287.17.2236

[4] Sloat BR, Cui Z. Nasal immunization with anthrax protective antigen protein adjuvanted with polyriboinosinic-polyribocytidylic acid induced strong mucosal and systemic immunities. Pharm Res. 2006;23(6):1217–26. 16718616 10.1007/s11095-006-0206-9

[5] Sloat BR, Shaker DS, Le UM, Cui Z. Nasal immunization with the mixture of PA63, LF, and a PGA conjugate induced strong antibody responses against all three antigens. FEMS Immunol Med Microbiol. 2008;52(2):169–79. 18194342 10.1111/j.1574-695X.2007.00347.x

[6] Klinman DM, Xie H, Little SF, Currie D, Ivins BE. CpG oligonucleotides improve the protective immune response induced by the anthrax vaccination of rhesus macaques. Vaccine. 2004;22(21–22):2881–6. 15246624 10.1016/j.vaccine.2003.12.020

[7] Donegan S, Bellamy R, Gamble CL. Vaccines for preventing anthrax. Cochrane Database Syst Rev. 2009;2009(2):Cd006403. 19370633 10.1002/14651858.CD006403.pub2 PMC6532564

[8] Cybulski RJ Jr, Sanz P, O’Brien AD. Anthrax vaccination strategies. Mol Aspects Med. 2009;30(6):490–502. 19729034 10.1016/j.mam.2009.08.006 PMC2783700

[9] Pittman PR, Gibbs PH, Cannon TL, Friedlander AM. Anthrax vaccine: short-term safety experience in humans. Vaccine. 2001;20(5–6):972–8. 11738765 10.1016/s0264-410x(01)00387-5

[10] Niu MT, Ball R, Woo EJ, Burwen DR, Knippen M, Braun MM. Adverse events after anthrax vaccination reported to the Vaccine Adverse Event Reporting System (VAERS), 1990-2007. Vaccine. 1990;27(2):290–7. 10.1016/j.vaccine.2008.10.044 18992783

[11] Vijaya Kumar K, Rudra A, Sreedhara MV, Siva Subramani T, Prasad DS, Das ML, et al. *Bacillus Calmette-Guérin* vaccine induces a selective serotonin reuptake inhibitor (SSRI)-resistant depression like phenotype in mice. Brain Behav Immun. 2014;42:204–11. 25016199 10.1016/j.bbi.2014.06.205

[12] O’Connor JC, Lawson MA, André C, Briley EM, Szegedi SS, Lestage J, et al. Induction of IDO by bacille Calmette-Guérin is responsible for development of murine depressive-like behavior. J Immunol. 2009;182(5):3202–12. 19234218 10.4049/jimmunol.0802722 PMC2664258

[13] Smith B, Leard CA, Smith TC, Reed RJ, Ryan MA. Anthrax vaccination in the Millennium Cohort: validation and measures of health. Am J Prev Med. 2007;32(4):347–53. 17383567 10.1016/j.amepre.2006.12.015

[14] Ciaffi J, Torrigiani G, Ruscitti P, Zambon A, Ursini F. No disproportionate reporting of inflammatory arthritis following COVID-19 vaccination: a pharmacovigilance study using VAERS data. Semin Arthritis Rheum. 2025;74:152827. 40939515 10.1016/j.semarthrit.2025.152827

[15] Alandijany TA, Qashqari FS. Evaluating the efficacy, safety, and immunogenicity of FDA-approved RSV vaccines: a systematic review of Arexvy, Abrysvo, and mResvia. Front Immunol. 2025;16:1624007. 40901480 10.3389/fimmu.2025.1624007 PMC12399520

[16] Manish M, Verma S, Kandari D, Kulshreshtha P, Singh S, Bhatnagar R. Anthrax prevention through vaccine and post-exposure therapy. Expert Opin Biol Ther. 2020;20(12):1405–25. 32729741 10.1080/14712598.2020.1801626

[17] Oliveira M, Marquez P, Ennulat C, Blanc P, Welsh K, Nair N, et al. Post-licensure safety surveillance of 20-valent pneumococcal conjugate vaccine (PCV20) among US adults in the vaccine adverse event reporting system (VAERS). Drug Saf. 2025;48(3):279–86. 39666166 10.1007/s40264-024-01498-2 PMC11973709

[18] Bonaldo G, Vaccheri A, D’Annibali O, Motola D. Safety profile of human papilloma virus vaccines: an analysis of the US Vaccine Adverse Event Reporting System from 2007 to 2017. Br J Clin Pharmacol. 2019;85(3):634–43. 30569481 10.1111/bcp.13841 PMC6379209

[19] Brown EG. Using MedDRA: implications for risk management. Drug Saf. 2004;27(8):591–602. 15154830 10.2165/00002018-200427080-00010

[20] Zhao K, Zhao Y, Xiao S, Tu C. Assessing the real-world safety of tralokinumab for atopic dermatitis: insights from a comprehensive analysis of FAERS data. Front Pharmacol. 2024;15:1458438. 39193341 10.3389/fphar.2024.1458438 PMC11347326

[21] Yu RJ, Krantz MS, Phillips EJ, Stone CA Jr. Emerging causes of drug-induced anaphylaxis: a review of anaphylaxis-associated reports in the FDA Adverse Event Reporting System (FAERS). J Allergy Clin Immunol Pract. 2021;9(2):819-29.e2. 32992044 10.1016/j.jaip.2020.09.021 PMC7870524

[22] Shu Y, Ding Y, Liu Y, Wu P, He X, Zhang Q. Post-marketing safety concerns with secukinumab: a disproportionality analysis of the FDA adverse event reporting system. Front Pharmacol. 2022;13:862508. 35754494 10.3389/fphar.2022.862508 PMC9214234

[23] Shabu A, Nishtala PS. Analysis of the adverse events following the mRNA-1273 COVID-19 vaccine. Expert Rev Vaccines. 2023;22(1):801–12. 37723099 10.1080/14760584.2023.2260477

[24] Qu H, Li W, Wu Z, Wang Y, Feng T, Li N, et al. Differences in hypersensitivity reactions and gadolinium deposition disease/symptoms associated with gadolinium exposure to gadolinium-based contrast agents: new insights based on global databases VigiBase, FAERS, and IQVIA-MIDAS. BMC Med. 2024;22(1):329. 39135199 10.1186/s12916-024-03537-2 PMC11321222

[25] Sessa M, Kragholm K, Hviid A, Andersen M. Thromboembolic events in younger women exposed to Pfizer-BioNTech or Moderna COVID-19 vaccines. Expert Opin Drug Saf. 2021;20(11):1451–3. 34264151 10.1080/14740338.2021.1955101 PMC8330010

[26] Sakaeda T, Tamon A, Kadoyama K, Okuno Y. Data mining of the public version of the FDA adverse event reporting system. Int J Med Sci. 2013;10(7):796–803. 23794943 10.7150/ijms.6048 PMC3689877

[27] Chen G, Li X, Sun M, Zhou Y, Yin M, Zhao B, et al. COVID-19 mRNA vaccines are generally safe in the short term: a vaccine vigilance real-world study says. Front Immunol. 2021;12:669010. 34093567 10.3389/fimmu.2021.669010 PMC8177815

[28] Nie W, Wu X, Xia Y, Zheng L, Lu H. Reported psychiatric adverse events among isotretinoin users: Monitoring priorities from a 20-year FDA Adverse Event Reporting System database study. J Am Acad Dermatol. 2025;93(1):64–72. 40024390 10.1016/j.jaad.2025.02.072

[29] Agodirin O, Olatoke S, Rahman G, Kolawole O, Oguntola S, Olasehinde O, et al. Determinants of late detection and advanced-stage diagnosis of breast cancer in Nigeria. PLoS ONE. 2021;16(11):e0256847. 34731161 10.1371/journal.pone.0256847 PMC8565753

[30] Li J, Liu S, Hu Y, Zhu L, Mao Y, Liu J. Predicting mortality in intensive care unit patients with heart failure using an interpretable machine learning model: retrospective cohort study. J Med Internet Res. 2022;24(8):e38082. 35943767 10.2196/38082 PMC9399880

[31] Edgar R, Domrachev M, Lash AE. Gene Expression Omnibus: NCBI gene expression and hybridization array data repository. Nucleic Acids Res. 2002;30(1):207–10. 11752295 10.1093/nar/30.1.207 PMC99122

[32] Klinman DM. CpG oligonucleotides accelerate and boost the immune response elicited by AVA, the licensed anthrax vaccine. Expert Rev Vaccines. 2006;5(3):365–9. 16827620 10.1586/14760584.5.3.365

[33] Xu M, Zhou H, Hu P, Pan Y, Wang S, Liu L, et al. Identification and validation of immune and oxidative stress-related diagnostic markers for diabetic nephropathy by WGCNA and machine learning. Front Immunol. 2023;14:1084531. 36911691 10.3389/fimmu.2023.1084531 PMC9992203

[34] Cheng M, Li M, Zhang Y, Gu X, Gao W, Zhang S, et al. Exploring the mechanism of PPCPs on human metabolic diseases based on network toxicology and molecular docking. Environ Int. 2025;196:109324. 39952201 10.1016/j.envint.2025.109324

[35] Chen L, Zhang YH, Wang S, Zhang Y, Huang T, Cai YD. Prediction and analysis of essential genes using the enrichments of gene ontology and KEGG pathways. PLoS ONE. 2017;12(9):e0184129. 28873455 10.1371/journal.pone.0184129 PMC5584762

[36] Wright JG, Quinn CP, Shadomy S, Messonnier N. Use of anthrax vaccine in the United States: recommendations of the Advisory Committee on Immunization Practices (ACIP), 2009. MMWR Recomm Rep. 2010;59(Rr-6):1–30. 20651644

[37] Stewart B, Zhang Y, Rose CE Jr, Tokars JI, Martin SW, Franzke LH, et al. Health-related quality of life in the Anthrax Vaccination Program for workers in the Laboratory Response Network. Vaccine. 2012;30(10):1841–6. 22230591 10.1016/j.vaccine.2011.12.128 PMC11325479

[38] Roser K, Mader L, Baenziger J, Sommer G, Kuehni CE, Michel G. Health-related quality of life in Switzerland: normative data for the SF-36v2 questionnaire. Qual Life Res. 2019;28(7):1963–77. 30848444 10.1007/s11136-019-02161-5 PMC6571102

[39] Gandon S, Lambert A, Voinson M, Day T, Parsons TL. The speed of vaccination rollout and the risk of pathogen adaptation. J R Soc Interface. 2025;22(228):20250060. 40628287 10.1098/rsif.2025.0060 PMC12308340

[40] Chu C, Artis D, Chiu IM. Neuro-immune interactions in the tissues. Immunity. 2020;52(3):464–74. 32187517 10.1016/j.immuni.2020.02.017 PMC10710744

[41] Udit S, Blake K, Chiu IM. Somatosensory and autonomic neuronal regulation of the immune response. Nat Rev Neurosci. 2022;23(3):157–71. 34997214 10.1038/s41583-021-00555-4 PMC9539447

[42] Sarapultsev A, Gusev E, Chereshnev V, Komelkova M, Hu D. Neuroimmune interactions in stress and depression: exploring the molecular and cellular mechanisms within the neuroinflammation-depression nexus. Curr Med Chem. 2024;33(13):2523–46. 10.2174/0109298673320710240920055041 39350557

[43] Gao Q, Hernandes MS. Sepsis-associated encephalopathy and blood-brain barrier dysfunction. Inflammation. 2021;44(6):2143–50. 34291398 10.1007/s10753-021-01501-3 PMC11044530

[44] Price RB, Duman R. Neuroplasticity in cognitive and psychological mechanisms of depression: an integrative model. Mol Psychiatry. 2020;25(3):530–43. 31801966 10.1038/s41380-019-0615-x PMC7047599

[45] Almqvist EW, Brinkman RR, Wiggins S, Hayden MR. Psychological consequences and predictors of adverse events in the first 5 years after predictive testing for Huntington’s disease. Clin Genet. 2003;64(4):300–9. 12974735 10.1034/j.1399-0004.2003.00157.x

[46] Bauer A, Knapp M, Alvi M, Chaudhry N, Gregoire A, Malik A, et al. Economic costs of perinatal depression and anxiety in a lower middle income country: Pakistan. J Affect Disord. 2024;357:60–7. 38642903 10.1016/j.jad.2024.04.061

[47] Lee JH, Meyer EJ, Nenke MA, Lightman SL, Torpy DJ. Cortisol, stress, and disease-bidirectional associations; role for corticosteroid-binding globulin? J Clin Endocrinol Metab. 2024;109(9):2161–72. 38941154 10.1210/clinem/dgae412

[48] Othman A, Sekheri M, Filep JG. Roles of neutrophil granule proteins in orchestrating inflammation and immunity. FEBS J. 2022;289(14):3932–53. 33683814 10.1111/febs.15803 PMC9546106

[49] Rajagopal C, Mains RE, Eipper BA. Signaling from the secretory granule to the nucleus. Crit Rev Biochem Mol Biol. 2012;47(4):391–406. 22681236 10.3109/10409238.2012.694845 PMC3920730

[50] Wu A, Zhang J. Neuroinflammation, memory, and depression: new approaches to hippocampal neurogenesis. J Neuroinflammation. 2023;20(1):283. 38012702 10.1186/s12974-023-02964-x PMC10683283

[51] Chavoshinezhad S, Beirami E, Izadpanah E. Neutrophils and NETosis in Alzheimer’s disease: Unraveling pathogenic mechanisms and novel therapeutic targets. Biomed Pharmacother. 2025;192:118568. 40972402 10.1016/j.biopha.2025.118568

[52] Ovsyannikova IG, Pankratz VS, Vierkant RA, Pajewski NM, Quinn CP, Kaslow RA, et al. Human leukocyte antigens and cellular immune responses to anthrax vaccine adsorbed. Infect Immun. 2013;81(7):2584–91. 23649091 10.1128/IAI.00269-13 PMC3697592

[53] Menard C, Pfau ML, Hodes GE, Kana V, Wang VX, Bouchard S, et al. Social stress induces neurovascular pathology promoting depression. Nat Neurosci. 2017;20(12):1752–60. 29184215 10.1038/s41593-017-0010-3 PMC5726568

[54] Hansen MB. Biomarkers of necrotising soft tissue infections aspects of the innate immune response. Dan Med J. 2017;64(7):B539. 28673381

[55] Xue D, Tabib T, Morse C, Yang Y, Domsic RT, Khanna D, et al. Expansion of Fcγ receptor IIIa-positive macrophages, ficolin 1-positive monocyte-derived dendritic cells, and plasmacytoid dendritic cells associated with severe skin disease in systemic sclerosis. Arthritis Rheumatol. 2022;74(2):329–41. 34042322 10.1002/art.41813 PMC8626521

[56] Mkrtchian A, Qiu Z, Abir Y, Erdmann T, Dercon Q, Sedlinska T, et al. Differential associations of dopamine and serotonin with reward and punishment processes in humans: a systematic review and meta-analysis. JAMA Psychiat. 2025;82(8):818–29. 10.1001/jamapsychiatry.2025.0839 PMC12159863 40498519

[57] Li S, Gao M, Mou Z, Zhang H, Wang Y, Zhang Y. Advances in neurotransmitter-mediated prefrontal circuitry in depression. Prog Neuropsychopharmacol Biol Psychiatry. 2025;141:111475. 40848830 10.1016/j.pnpbp.2025.111475

[58] Shi Z, Guan N, Sun W, Sun T, Niu L, Li J, et al. Protective effect of *Levilactobacillus brevis* against *Yersinia enterocolitica* infection in mouse model via regulating MAPK and NF-κB pathway. Probiotics Antimicrob Proteins. 2022;14(5):830–44. 35665480 10.1007/s12602-022-09957-x

[59] Raymond B, Ravaux L, Mémet S, Wu Y, Sturny-Leclère A, Leduc D, et al. Anthrax lethal toxin down-regulates type-IIA secreted phospholipase A(2) expression through MAPK/NF-kappaB inactivation. Biochem Pharmacol. 2010;79(8):1149–55. 19962969 10.1016/j.bcp.2009.11.023

[60] Yan M, Roehrl MH, Basar E, Wang JY. Selection and evaluation of the immunogenicity of protective antigen mutants as anthrax vaccine candidates. Vaccine. 2008;26(7):947–55. 18192092 10.1016/j.vaccine.2007.11.087 PMC2254513

[61] Modi T, Gervais D, Smith S, Miller J, Subramaniam S, Thalassinos K, et al. Characterization of the UK anthrax vaccine and human immunogenicity. Hum Vaccin Immunother. 2021;17(3):747–58. 32897798 10.1080/21645515.2020.1799668 PMC7993152

[62] Grignoli N, Petrocchi S, Polito A, Gagliano V, Sallusto F, Uguccioni M, et al. The interplay between previous infection and mental health condition on antibody response to COVID-19 mRNA vaccination. Brain Behav Immun Health. 2023;33:100677. 37701787 10.1016/j.bbih.2023.100677 PMC10493882

[63] Agbadamashi DJ, Price CL. Novel strategies for preventing fungal infections-outline. Pathogens. 2025. 10.3390/pathogens14020126. 40005503 10.3390/pathogens14020126 PMC11858109

[64] Keller H. Comparison of the adverse effect profile of different substances such as penicillins, tetracyclines, sulfonamides and quinolones. Infection. 1991;19(Suppl 1):S19-24. 2007510 10.1007/BF01644730

[65] He K, He L, Wei X, Wang X, Zhou M, Cao Y, et al. Levofloxacin-induced seizure susceptibility involves both enhanced glutamatergic and impaired GABAergic synaptic function. Brain Res. 2025;1866:149929. 40915475 10.1016/j.brainres.2025.149929

[66] Shabel SJ, Proulx CD, Piriz J, Malinow R. Mood regulation GABA/glutamate co-release controls habenula output and is modified by antidepressant treatment. Science. 2014;345(6203):1494–8. 25237099 10.1126/science.1250469 PMC4305433

[67] Sun H, Yang B, Zhu X, Li Q, Song E, Song Y. Oral exposure of polystyrene microplastics and doxycycline affects mice neurological function via gut microbiota disruption: The orchestrating role of fecal microbiota transplantation. J Hazard Mater. 2024;467:133714. 38340564 10.1016/j.jhazmat.2024.133714

[68] Chu VT, Glascock A, Donnell D, Grabow C, Brown CE, Ward R, et al. Impact of doxycycline post-exposure prophylaxis for sexually transmitted infections on the gut microbiome and antimicrobial resistome. Nat Med. 2025;31(1):207–17. 39363100 10.1038/s41591-024-03274-2 PMC11750720

[69] Sun P, Wang M, Liu YX, Li L, Chai X, Zheng W, et al. High-fat diet-disturbed gut microbiota-colonocyte interactions contribute to dysregulating peripheral tryptophan-kynurenine metabolism. Microbiome. 2023;11(1):154. 37468922 10.1186/s40168-023-01606-x PMC10355067

[70] Walker AK, Wing EE, Banks WA, Dantzer R. Leucine competes with kynurenine for blood-to-brain transport and prevents lipopolysaccharide-induced depression-like behavior in mice. Mol Psychiatry. 2019;24(10):1523–32. 29988087 10.1038/s41380-018-0076-7 PMC6326900

[71] Gao W, Yu J, Sun Y, Song Z, Liu X, Han X, et al. Adverse events in the nervous system associated with blinatumomab: a real-world study. BMC Med. 2025;23(1):72. 39915776 10.1186/s12916-025-03913-6 PMC11804067

[72] Aksar A, Lutz J, Wagner E, Strube W, Luykx JJ, Hasan A. Vaccination and clozapine use: a systematic review and an analysis of the VAERS database. Eur Arch Psychiatry Clin Neurosci. 2025;275(1):141–62. 38165458 10.1007/s00406-023-01729-0

[73] Suares JE, Khan S, Aadrika A, Poojari PG, Rashid M, Thunga G. Vaccine-associated paralytic poliomyelitis in oral polio vaccine recipients: disproportionality analysis using VAERS and systematic review. Expert Opin Drug Saf. 2024;23(7):855–67. 38813942 10.1080/14740338.2024.2359616

