# Supplementary material for: Real-world analysis of depression-related adverse events associated with the adsorbed anthrax vaccine: integrating pharmacovigilance signals, machine learning risk prediction, and transcriptomic mechanisms
Source: Infect Dis Poverty. 2026 Aug 10;15:86. doi: 10.1186/s40249-026-01483-0 (PMC13455343; doi:10.1186/s40249-026-01483-0)
Supplement: Supplementary file 1 [file 40249_2026_1483_MOESM1_ESM.docx]

**Supplementary Table 1:** Ratio imbalance measurement algorithm.

| **Characteristics** | **AEs of interest** | **Other AEs** | **Total** |
| --- | --- | --- | --- |
| **Target drug** | a | b | a + b |
| **Other drugs** | c | d | c + d |
| **Total** | a + c | b + d | N = a + b + c + d |

a: Number of reports that contain both targeted vaccine and targeted vaccine adverse reactions; b: Number of reports of other vaccine adverse reactions that contain the targeted vaccine; c: Number of reports of targeted vaccine adverse reactions that contain other vaccines; d: Number of reports that contain other vaccines and other vaccine adverse reactions; All other vaccines, all vaccines in the VAERS, dataset other than AVA.

**Supplementary Table 2:** Summary of major algorithms used for signal detection.

| **Method** | **Formula** | **Threshold** |
| --- | --- | --- |
| **ROR** | ROR = ad/b/c  95%CI = e ^ln (ROR)±1.96(l/a+1/b+l/c+l/d) ^0.5^ | a ≥ 3  ROR ≥ 3  95%CI (lower limit) > 1 |
| **PRR** | PRR = a (c + d)/c/(a+b)  x^2^ = [(ad-bc) ^2] (a+b + c + d)/[(a+b) (c + d) (a+c) (b + d) | a ≥ 3  PRR ≥ 2  95%CI (lower limit) > 1 |
| **BCPNN** | IC = logza (a+b + c + d)/((a+c) (a+b))  95%Cl = E(IC)±2V(IC) ^0.5 | IC025 > 0 |
| **MGPS** | EBGM = a (a+b + c + d)/(a+c)/(a+b)  95%CI = e ^ln (EBGM)±1.96(l/a+l/b+l/c+l/d) ^0.5^ | EBGM05 > 2 |

ROR: reporting odds ratio; PRR, proportional reporting ratio; BCPNN: bayesian confidence propagation neural network; MGPS: Multi-item gamma Poisson shrinker; 95% CI: 95% confidence interval; EBGM: empirical Bayesian geometric mean.

**Supplementary Table 3: Criteria and relevant scores to prioritize AEs emerged from disproportionality analysis.**

| **Criterium** | **2 points** | **1 point** | | **0 point** |
| --- | --- | --- | --- | --- |
| **Reporting rate (cases/non-cases)** | > 10% | 1–10% | | 0–1% |
| **Signal stability (consistency across disproportionality analyses)** | 3 of 3 | | 2 of 3 | 1 of 3 |
| **Reported case fatality rate (proportion of reports with death as outcome)** | > 50% | | 25–50% | < 25% |
| **Clinical relevance (serious likely drug-attributable AEs)** | DME | | IME | None |

DME: designated medical event, IME: important medical event.

**Supplementary Table 4:**

**Characteristics of anthrax vaccines from VAERS between 1990 and 2025.**

| **Characteristics** | **Case number** | **Proportion (%)** |
| --- | --- | --- |
| **Total vaccines** | 9446 |  |
| **Vaccine type** |  |  |
| **ANTH** | 9446 | 100 |
| **Vaccine name** |  |  |
| **ANTHRAX (BIOTHRAX)** | 6533 | 69.2 |
| **ANTHRAX (CYFENDUS)** | 8 | 0.1 |
| **ANTHRAX (NO BRAND NAME)** | 2905 | 30.8 |
| **Administration dose** |  |  |
| **1** | 2014 | 21.3 |
| **2** | 1516 | 16.0 |
| **3** | 1229 | 13.0 |
| **4** | 1010 | 10.7 |
| **5** | 805 | 8.5 |
| **6** | 544 | 5.8 |
| **7+** | 577 | 6.1 |
| **N/A** | 4 | 0.1 |
| **UNK** | 1747 | 18.5 |
| **Vaccination routes** |  |  |
| **Absorption (unspecified)** | 36 | 0.4 |
| **Intradermal** | 73 | 0.8 |
| **Intravenous** | 30 | 0.3 |
| **Intramuscular** | 2345 | 24.8 |
| **Other** | 19 | 0.2 |
| **Subcutaneous** | 3924 | 41.5 |
| **Subcutaneous (Abdomen)** | 25 | 0.3 |
| **Subcutaneous (Leg)** | 1 | 0 |
| **Syringe** | 195 | 2.1 |
| **Unknown** | 1049 | 11.1 |
| **Missing** | 1749 | 18.5 |
| **Vaccination site** |  |  |
| **Arm (unspecified)** | 84 | 0.9 |
| **Gluteal Muscle** | 2 | 0 |
| **Left Arm** | 3078 | 32.6 |
| **Left Leg** | 19 | 0.2 |
| **Other** | 3 | 0 |
| **Right Arm** | 2667 | 28.2 |
| **Right Leg** | 20 | 0.2 |
| **Unknown** | 1515 | 16.0 |
| **Missing** | 2058 | 21.8 |

**Supplementary Table 5:**

**Clinical characteristics of depression-related AEs in males injected with anthrax vaccine from the VAERS Database.**

| **Characteristics** | **Case number** | **Proportion (%)** |
| --- | --- | --- |
| **Number of events** | 140 |  |
| **Gender (%)** |  |  |
| **Male** | 140 | 100 |
| **Age(years)** |  |  |
| **18~65** | 133 | 95.0 |
| **Not specified** | 7 | 5.0 |
| **Reported States** |  |  |
| **Puerto Rico** | 68 | 48.6 |
| **Virginia** | 6 | 4.3 |
| **Texas** | 6 | 4.3 |
| **California** | 5 | 3.6 |
| **Carolina** | 5 | 3.6 |
| **others** | 50 | 35.6 |
| **Serious AEs** |  |  |
| **No** | 73 | 52.1 |
| **Yes** | 67 | 47.9 |
| **Single Vaccine Injection** |  |  |
| **No** | 49 | 35.0 |
| **Yes** | 91 | 65.0 |
| **Outcome** |  |  |
| **Death** | 1 | 0.7 |
| **Life threatening** | 28 | 20.0 |
| **Hospitalization** | 25 | 17.9 |
| **Prolonged hospitalization** | 3 | 2.1 |
| **Disability** | 47 | 33.6 |
| **Recovered** | 6 | 4.3 |
| **Not available** | 30 | 21.4 |

**Supplementary Table 6:**

**Clinical characteristics of depression-related AEs in females injected with anthrax vaccine from the VAERS Database.**

| **Characteristics** | **Case number** | **Proportion (%)** |
| --- | --- | --- |
| **Number of events** | 31 |  |
| **Gender (%)** |  |  |
| **Female** | 31 | 100 |
| **Age(years)** |  |  |
| **18~65** | 29 | 93.5 |
| **Not specified** | 2 | 6.5 |
| **Reported States** |  |  |
| **Puerto Rico** | 6 | 19.4 |
| **Virginia** | 3 | 9.7 |
| **California** | 2 | 6.5 |
| **Missouri** | 2 | 6.5 |
| **others** | 18 | 57.9 |
| **Serious AEs** |  |  |
| **No** | 19 | 61.3 |
| **Yes** | 12 | 38.7 |
| **Single Vaccine Injection** |  |  |
| **No** | 11 | 35.5 |
| **Yes** | 20 | 64.5 |
| **Outcome** |  |  |
| **Death** | 0 | 0 |
| **Life threatening** | 4 | 12.9 |
| **Hospitalization** | 6 | 19.4 |
| **Prolonged hospitalization** | 0 | 0 |
| **Disability** | 7 | 22.6 |
| **Recovered** | 4 | 12.9 |
| **Not available** | 10 | 32.3 |

**Supplementary Table 7:**

**Clinical characteristics of depression-related AEs in patients aged 18-30 injected with anthrax vaccine from the VAERS Database.**

| **Characteristics** | **Case number** | **Proportion (%)** |
| --- | --- | --- |
| **Number of events** | 46 |  |
| **Gender (%)** |  |  |
| **Female** | 14 | 30.4 |
| **Male** | 31 | 67.4 |
| **Not specified** | 1 | 2.2 |
| **Age(years)** |  |  |
| **<18** | 0 | 0 |
| **18∼65** | 46 | 100 |
| **Reported States** |  |  |
| **Puerto Rico** | 9 | 19.6 |
| **Virginia** | 7 | 15.2 |
| **California** | 4 | 8.7 |
| **Texas** | 3 | 6.5 |
| **others** | 23 | 50 |
| **Serious AEs** |  |  |
| **No** | 21 | 45.7 |
| **Yes** | 25 | 54.3 |
| **Single Vaccine Injection** |  |  |
| **No** | 19 | 41.3 |
| **Yes** | 27 | 58.7 |
| **Outcome** |  |  |
| **Death** | 0 | 0 |
| **Life threatening** | 10 | 21.7 |
| **Hospitalization** | 16 | 34.8 |
| **Prolonged hospitalization** | 1 | 2.2 |
| **Disability** | 17 | 37 |
| **Recovered** | 2 | 4.3 |
| **Not available** | 0 | 0 |

**Supplementary Table 8:**

**Clinical characteristics of depression-related AEs in patients aged 30-45 injected with anthrax vaccine from the VAERS Database.**

| **Characteristics** | **Case number** | **Proportion (%)** |
| --- | --- | --- |
| **Number of events** | 87 |  |
| **Gender (%)** |  |  |
| **Female** | 14 | 30.4 |
| **Male** | 31 | 67.4 |
| **Not specified** | 1 | 2.2 |
| **Age(years)** |  |  |
| **<18** | 0 | 0 |
| **18∼65** | 46 | 100 |
| **Reported States** |  |  |
| **Puerto Rico** | 9 | 19.6 |
| **Virginia** | 7 | 15.2 |
| **California** | 4 | 8.7 |
| **Texas** | 3 | 6.5 |
| **others** | 23 | 50 |
| **Serious AEs** |  |  |
| **No** | 21 | 45.7 |
| **Yes** | 25 | 54.3 |
| **Single Vaccine Injection** |  |  |
| **No** | 19 | 41.3 |
| **Yes** | 27 | 58.7 |
| **Outcome** |  |  |
| **Death** | 1 | 1.1 |
| **Life threatening** | 13 | 14.9 |
| **Hospitalization** | 12 | 13.8 |
| **Prolonged hospitalization** | 2 | 2.3 |
| **Disability** | 26 | 29.9 |
| **Recovered** | 6 | 6.9 |
| **Not available** | 27 | 31.1 |

**Supplementary Table 9:**

**Clinical characteristics of depression-related AEs in patients aged 45-65 injected with anthrax vaccine from the VAERS Database.**

| **Characteristics** | **Case number** | **Proportion (%)** |
| --- | --- | --- |
| **Number of events** | 33 |  |
| **Gender (%)** |  |  |
| **Female** | 1 | 3.0 |
| **Male** | 30 | 90.9 |
| **Not specified** | 2 | 6.1 |
| **Reported States** |  |  |
| **Puerto Rico** | 25 | 75.8 |
| **South Dakota** | 1 | 3.0 |
| **New Mexico** | 1 | 3.0 |
| **North Carolina** | 1 | 3.0 |
| **others** | 5 | 15.2 |
| **Serious AEs** |  |  |
| **No** | 16 | 48.5 |
| **Yes** | 17 | 51.5 |
| **Single Vaccine Injection** |  |  |
| **No** | 11 | 33.3 |
| **Yes** | 22 | 66.7 |
| **Outcome** |  |  |
| **Death** | 0 | 0 |
| **Life threatening** | 8 | 24.2 |
| **Hospitalization** | 3 | 9.1 |
| **Prolonged hospitalization** | 0 | 0 |
| **Disability** | 10 | 30.3 |
| **Recovered** | 1 | 3.0 |
| **Not available** | 27 | 33.4 |

**Supplementary Table 10:** PTs of Depression-Related AEs Following Single-Injection of AVA Vaccine: Signal Strength from the VAERS Database.

| **Preferred terms (PTs)** | **Case reports** | **ROR (95% CI)** | | **PRR (χ²)** | **EBGM**  **(EBGM05)** | **IC(IC025)** |
| --- | --- | --- | --- | --- | --- | --- |
| **Depression*** | 119 | | 4.97 ( 4.14 - 5.96 ) | 4.95 ( 370.21 ) | 4.9 ( 4.21 ) | 2.29 ( 2.03 ) |

Asterisks (*) indicate statistically significant signals. Abbreviations: ROR, reporting odds ratio; PRR, proportional reporting ratio; EBGM, empirical Bayesian geometric mean; EBGM05, the lower limit of the 95% confidence interval of EBGM; IC, information component; IC025, the lower limit of the 95% confidence interval of the IC.

**Supplementary Table 11:** **Performance Metrics of Different Machine Learning Models**

| **Evaluation Metric** | **LASSO Logistic Regression** | **K-Nearest Neighbors (KNN)** | **Support Vector Machine (SVM)** | **Random Forest** | **Naive Bayes** | **XGBoost** | **Decision Tree** |
| --- | --- | --- | --- | --- | --- | --- | --- |
| **Sensitivity** | 0 | 0.013333 | 0 | 0.213333 | 0.96 | 0.253333 | 0.08 |
| **Specificity** | 0.99855 | 0.999275 | 1 | 0.99855 | 0.176577 | 0.995286 | 0.997825 |
| **Positive Predictive Value (PPV)** | 0 | 0.333333 |  | 0.8 | 0.03073 | 0.59375 | 0.5 |
| **Negative Predictive Value (NPV)** | 0.973489 | 0.973852 | 0.973526 | 0.979026 | 0.993878 | 0.980007 | 0.975541 |
| **Precision** | 0 | 0.333333 |  | 0.8 | 0.03073 | 0.59375 | 0.5 |
| **Recall** | 0 | 0.013333 | 0 | 0.213333 | 0.96 | 0.253333 | 0.08 |
| **F1 Score** |  | 0.025641 |  | 0.336842 | 0.059553 | 0.35514 | 0.137931 |
| **Balanced Accuracy** | 0.499275 | 0.506304 | 0.5 | 0.605942 | 0.568289 | 0.62431 | 0.538912 |
| **Accuracy** | 0.972114 | 0.973173 | 0.973526 | 0.977762 | 0.197317 | 0.975644 | 0.973526 |

**Supplementary Table 15:**

**Clinical Characteristics of Depression-Related AEs in Patients Treated with drugs (unspecific) from the FAERS Database.**

| **Characteristics** | **Case number** | **Proportion (%)** |
| --- | --- | --- |
| **Number of events** | 1763 |  |
| **Gender (%)** |  |  |
| **Female** | 998 | 56.6 |
| **Male** | 667 | 38.4 |
| **Not specified** | 88 | 5.0 |
| **Weight (kg)** |  |  |
| **<50** | 94 | 5.3 |
| **>100** | 96 | 5.4 |
| **50∼100** | 1018 | 57.7 |
| **Not specified** | 555 | 31.5 |
| **Age(years)** |  |  |
| **<18** | 43 | 2.4 |
| **18∼65** | 1266 | 71.8 |
| **65∼85** | 171 | 9.7 |
| **＞85** | 16 | 0.9 |
| **Not specified** | 267 | 15.1 |
| **Reported Countries** |  |  |
| **United Kingdom** | 601 | 34.1 |
| **United States** | 560 | 31.7 |
| **Germany** | 264 | 14.9 |
| **Canada** | 122 | 6.9 |
| **Netherlands** | 60 | 3.2 |
| **others** | 156 | 9.2 |
| **Reporter** |  |  |
| **Consumer** | 1043 | 59.2 |
| **Physician** | 165 | 9.4 |
| **Health Professional** | 151 | 8.6 |
| **Pharmacist** | 32 | 1.8 |
| **Not specified** | 372 | 21.1 |
| **Year of report** |  |  |
| **2004** | 2 | 0.1 |
| **2005** | 0 | 0 |
| **2006** | 10 | 0.6 |
| **2007** | 12 | 0.7 |
| **2008** | 17 | 1.0 |
| **2009** | 17 | 1.0 |
| **2010** | 16 | 0.9 |
| **2011** | 30 | 1.7 |
| **2012** | 33 | 1.9 |
| **2013** | 46 | 2.6 |
| **2014** | 81 | 4.6 |
| **2015** | 88 | 5.0 |
| **2016** | 167 | 9.5 |
| **2017** | 141 | 8.0 |
| **2018** | 116 | 6.6 |
| **2019** | 226 | 12.8 |
| **2020** | 160 | 9.1 |
| **2021** | 144 | 8.2 |
| **2022** | 70 | 4.0 |
| **2023** | 181 | 10.3 |
| **2024** | 157 | 8.9 |
| **2025** | 49 | 2.8 |
| **Outcomes** |  |  |
| **Death** | 48 | 2.7 |
| **Other serious outcome** | 912 | 51.7 |
| **Hospitalization** | 240 | 13.6 |
| **Life-Threatening** | 108 | 6.1 |
| **Disability** | 454 | 25.8 |

**Supplementary Table 16:**

**Clinical Characteristics of Depression-Related AEs in Patients Treated with Levofloxacin from the FAERS Database.**

| **Characteristics** | **Case number** | **Proportion (%)** |
| --- | --- | --- |
| **Number of events** | 616 |  |
| **Gender (%)** |  |  |
| **Female** | 326 | 52.9 |
| **Male** | 219 | 35.6 |
| **Not specified** | 71 | 11.5 |
| **Weight (kg)** |  |  |
| **<50** | 24 | 3.9 |
| **>100** | 41 | 6.7 |
| **50∼100** | 287 | 46.6 |
| **Not specified** | 264 | 42.9 |
| **Age(years)** |  |  |
| **<18** | 5 | 0.8 |
| **18∼65** | 370 | 60.1 |
| **65∼85** | 89 | 14.4 |
| **＞85** | 6 | 1.0 |
| **Not specified** | 146 | 23.7 |
| **Reported Countries** |  |  |
| **United States** | 305 | 49.5 |
| **Germany** | 133 | 21.6 |
| **United Kingdom** | 52 | 8.4 |
| **Canada** | 25 | 4.1 |
| **France** | 24 | 3.9 |
| **others** | 77 | 12.5 |
| **Reporter** |  |  |
| **Consumer** | 400 | 64.9 |
| **Physician** | 50 | 8.1 |
| **Health Professional** | 34 | 5.5 |
| **Pharmacist** | 12 | 1.9 |
| **Not specified** | 120 | 19.5 |
| **Year of report** |  |  |
| **2004** | 1 | 0.2 |
| **2005** | 0 | 0 |
| **2006** | 1 | 0.2 |
| **2007** | 2 | 0.3 |
| **2008** | 1 | 0.2 |
| **2009** | 0 | 0 |
| **2010** | 2 | 0.3 |
| **2011** | 5 | 0.8 |
| **2012** | 11 | 1.8 |
| **2013** | 7 | 1.1 |
| **2014** | 38 | 6.2 |
| **2015** | 75 | 12.2 |
| **2016** | 81 | 13.1 |
| **2017** | 61 | 9.9 |
| **2018** | 59 | 9.6 |
| **2019** | 75 | 12.2 |
| **2020** | 47 | 7.6 |
| **2021** | 39 | 6.3 |
| **2022** | 16 | 2.6 |
| **2023** | 44 | 7.1 |
| **2024** | 31 | 5.0 |
| **2025** | 20 | 3.2 |
| **Outcomes** |  |  |
| **Death** | 15 | 2.4 |
| **Other serious outcome** | 247 | 40.1 |
| **Hospitalization** | 136 | 22.1 |
| **Life-Threatening** | 49 | 8.0 |
| **Disability** | 247 | 40.1 |

**Supplementary Table 17:**

**Clinical Characteristics of Depression-Related AEs in Patients Treated with Doxycycline from the FAERS Database.**

| **Characteristics** | **Case number** | **Proportion (%)** |
| --- | --- | --- |
| **Number of events** | 313 |  |
| **Gender (%)** |  |  |
| **Female** | 184 | 58.8 |
| **Male** | 102 | 32.6 |
| **Not specified** | 27 | 8.6 |
| **Weight (kg)** |  |  |
| **<50** | 18 | 5.8 |
| **>100** | 16 | 5.1 |
| **50∼100** | 167 | 53.4 |
| **Not specified** | 112 | 35.8 |
| **Age(years)** |  |  |
| **<18** | 31 | 9.9 |
| **18∼65** | 209 | 66.8 |
| **65∼85** | 18 | 5.8 |
| **＞85** | 0 | 0 |
| **Not specified** | 55 | 17.6 |
| **Reported Countries** |  |  |
| **United States** | 98 | 31.3 |
| **United Kingdom** | 170 | 54.4 |
| **Canada** | 10 | 3.2 |
| **Australia** | 6 | 1.9 |
| **Netherlands** | 6 | 1.9 |
| **others** | 23 | 7.3 |
| **Reporter** |  |  |
| **Consumer** | 173 | 55.3 |
| **Physician** | 46 | 14.7 |
| **Health Professional** | 32 | 10.2 |
| **Pharmacist** | 4 | 1.3 |
| **Not specified** | 58 | 18.5 |
| **Year of report** |  |  |
| **2004** | 0 | 0 |
| **2005** | 0 | 0 |
| **2006** | 0 | 0 |
| **2007** | 1 | 0.3 |
| **2008** | 8 | 2.6 |
| **2009** | 9 | 2.9 |
| **2010** | 3 | 1.0 |
| **2011** | 1 | 0.3 |
| **2012** | 2 | 0.6 |
| **2013** | 5 | 1.6 |
| **2014** | 13 | 4.2 |
| **2015** | 21 | 6.7 |
| **2016** | 14 | 4.5 |
| **2017** | 27 | 8.6 |
| **2018** | 15 | 4.8 |
| **2019** | 34 | 10.9 |
| **2020** | 21 | 6.7 |
| **2021** | 15 | 4.8 |
| **2022** | 17 | 5.4 |
| **2023** | 33 | 10.5 |
| **2024** | 61 | 19.5 |
| **2025** | 13 | 4.2 |
| **Outcomes** |  |  |
| **Death** | 9 | 2.9 |
| **Other serious outcome** | 208 | 66.5 |
| **Hospitalization** | 27 | 8.6 |
| **Life-Threatening** | 21 | 6.7 |
| **Disability** | 48 | 15.3 |

**Supplementary Table 18:**

**Clinical Characteristics of Depression-Related AEs in Patients Treated with Moxifloxacin from the FAERS Database.**

| **Characteristics** | **Case number** | **Proportion (%)** |
| --- | --- | --- |
| **Number of events** | 144 |  |
| **Gender (%)** |  |  |
| **Female** | 93 | 64.6 |
| **Male** | 46 | 31.9 |
| **Not specified** | 5 | 3.5 |
| **Weight (kg)** |  |  |
| **<50** | 4 | 2.8 |
| **>100** | 9 | 6.3 |
| **50∼100** | 72 | 50.0 |
| **Not specified** | 59 | 41.0 |
| **Age(years)** |  |  |
| **<18** | 0 | 0 |
| **18∼65** | 87 | 60.4 |
| **65∼85** | 29 | 20.1 |
| **＞85** | 1 | 0.7 |
| **Not specified** | 27 | 18.8 |
| **Reported Countries** |  |  |
| **United States** | 52 | 36.1 |
| **Canada** | 35 | 24.3 |
| **Germany** | 19 | 13.2 |
| **China** | 11 | 7.6 |
| **Türkiye** | 5 | 3.5 |
| **others** | 22 | 15.3 |
| **Reporter** |  |  |
| **Consumer** | 65 | 45.1 |
| **Physician** | 17 | 11.8 |
| **Health Professional** | 14 | 9.7 |
| **Pharmacist** | 5 | 3.5 |
| **Not specified** | 43 | 29.9 |
| **Year of report** |  |  |
| **2004** | 1 | 0.7 |
| **2005** | 1 | 0.7 |
| **2006** | 0 | 0 |
| **2007** | 0 | 0 |
| **2008** | 1 | 0.7 |
| **2009** | 0 | 0 |
| **2010** | 1 | 0.7 |
| **2011** | 2 | 1.4 |
| **2012** | 1 | 0.7 |
| **2013** | 0 | 0 |
| **2014** | 2 | 1.4 |
| **2015** | 7 | 4.9 |
| **2016** | 23 | 16.0 |
| **2017** | 18 | 12.5 |
| **2018** | 20 | 13.9 |
| **2019** | 19 | 13.2 |
| **2020** | 9 | 6.3 |
| **2021** | 6 | 4.2 |
| **2022** | 6 | 4.2 |
| **2023** | 7 | 4.9 |
| **2024** | 14 | 9.7 |
| **2025** | 6 | 4.2 |
| **Outcomes** |  |  |
| **Death** | 4 | 2.8 |
| **Other serious outcome** | 75 | 52.1 |
| **Hospitalization** | 30 | 20.8 |
| **Life-Threatening** | 14 | 9.7 |
| **Disability** | 21 | 14.6 |

**Supplementary Table 19:**

**Clinical Characteristics of Depression-Related AEs in Patients Treated with Minocycline from the FAERS Database.**

| **Characteristics** | **Case number** | **Proportion (%)** |
| --- | --- | --- |
| **Number of events** | 36 |  |
| **Gender (%)** |  |  |
| **Female** | 22 | 61.1 |
| **Male** | 12 | 33.3 |
| **Not specified** | 2 | 5.6 |
| **Weight (kg)** |  |  |
| **<50** | 7 | 19.4 |
| **>100** | 0 | 0 |
| **50∼100** | 17 | 47.2 |
| **Not specified** | 12 | 33.3 |
| **Age(years)** |  |  |
| **<18** | 8 | 22.2 |
| **18∼65** | 19 | 52.8 |
| **65∼85** | 1 | 2.8 |
| **＞85** | 0 | 0 |
| **Not specified** | 8 | 22.2 |
| **Reported Countries** |  |  |
| **United States** | 25 | 69.4 |
| **Germany** | 3 | 8.3 |
| **United Kingdom** | 2 | 5.6 |
| **Canada** | 1 | 2.8 |
| **Brazil** | 1 | 2.8 |
| **others** | 4 | 11.1 |
| **Reporter** |  |  |
| **Consumer** | 15 | 41.7 |
| **Physician** | 7 | 19.4 |
| **Health Professional** | 3 | 8.3 |
| **Pharmacist** | 2 | 5.6 |
| **Not specified** | 9 | 25.0 |
| **Year of report** |  |  |
| **2004** | 0 | 0 |
| **2005** | 1 | 2.8 |
| **2006** | 0 | 0 |
| **2007** | 1 | 2.8 |
| **2008** | 0 | 0 |
| **2009** | 0 | 0 |
| **2010** | 3 | 8.3 |
| **2011** | 0 | 0 |
| **2012** | 2 | 5.6 |
| **2013** | 1 | 2.8 |
| **2014** | 1 | 2.8 |
| **2015** | 3 | 8.3 |
| **2016** | 7 | 19.4 |
| **2017** | 3 | 8.3 |
| **2018** | 1 | 2.8 |
| **2019** | 2 | 5.6 |
| **2020** | 0 | 0 |
| **2021** | 5 | 13.9 |
| **2022** | 0 | 0 |
| **2023** | 0 | 0 |
| **2024** | 6 | 16.7 |
| **2025** | 0 | 0 |
| **Outcomes** |  |  |
| **Death** | 1 | 2.8 |
| **Other serious outcome** | 25 | 69.4 |
| **Hospitalization** | 4 | 11.1 |
| **Life-Threatening** | 5 | 13.9 |
| **Disability** | 1 | 2.8 |

**Supplementary Table 20:**

**Clinical Characteristics of Depression-Related AEs in Patients Treated with Penicillin from the FAERS Database.**

| **Characteristics** | **Case number** | **Proportion (%)** |
| --- | --- | --- |
| **Number of events** | 7 |  |
| **Gender (%)** |  |  |
| **Female** | 4 | 57.1 |
| **Male** | 3 | 42.9 |
| **Not specified** | 0 | 0 |
| **Weight (kg)** |  |  |
| **<50** | 1 | 14.3 |
| **>100** | 0 | 0 |
| **50∼100** | 2 | 28.6 |
| **Not specified** | 4 | 57.1 |
| **Age(years)** |  |  |
| **<18** | 0 | 0 |
| **18∼65** | 6 | 85.7 |
| **65∼85** | 1 | 14.3 |
| **＞85** | 0 | 0 |
| **Not specified** | 0 | 0 |
| **Reported Countries** |  |  |
| **United States** | 3 | 52.8 |
| **United Kingdom** | 2 | 28.6 |
| **others** | 2 | 28.6 |
| **Reporter** |  |  |
| **Consumer** | 2 | 28.6 |
| **Physician** | 3 | 42.9 |
| **Health Professional** | 0 | 0 |
| **Pharmacist** | 0 | 0 |
| **Not specified** | 2 | 28.6 |
| **Year of report** |  |  |
| **2004** | 0 | 0 |
| **2005** | 0 | 0 |
| **2006** | 0 | 0 |
| **2007** | 0 | 0 |
| **2008** | 1 | 14.3 |
| **2009** | 0 | 0 |
| **2010** | 0 | 0 |
| **2011** | 1 | 14.3 |
| **2012** | 0 | 0 |
| **2013** | 0 | 0 |
| **2014** | 0 | 0 |
| **2015** | 0 | 0 |
| **2016** | 0 | 0 |
| **2017** | 0 | 0 |
| **2018** | 0 | 0 |
| **2019** | 1 | 14.3 |
| **2020** | 1 | 14.3 |
| **2021** | 1 | 14.3 |
| **2022** | 0 | 0 |
| **2023** | 0 | 0 |
| **2024** | 2 | 28.6 |
| **2025** | 0 | 0 |
| **Outcomes** |  |  |
| **Death** | 0 | 0 |
| **Other serious outcome** | 5 | 71.5 |
| **Hospitalization** | 1 | 14.3 |
| **Life-Threatening** | 0 | 0 |
| **Disability** | 1 | 14.3 |

**Supplementary Table 21:**

**Clinical Characteristics of Depression-Related AEs in Patients Treated with Ceftriaxone from the FAERS Database.**

| **Characteristics** | **Case number** | **Proportion (%)** |
| --- | --- | --- |
| **Number of events** | 4 |  |
| **Gender (%)** |  |  |
| **Female** | 4 | 100 |
| **Male** | 0 | 0 |
| **Not specified** | 0 | 0 |
| **Weight (kg)** |  |  |
| **<50** | 0 | 0 |
| **>100** | 0 | 0 |
| **50∼100** | 0 | 0 |
| **Not specified** | 4 | 100 |
| **Age(years)** |  |  |
| **<18** | 0 | 0 |
| **18∼65** | 2 | 50.0 |
| **65∼85** | 0 | 0 |
| **＞85** | 0 | 0 |
| **Not specified** | 2 | 50.0 |
| **Reported Countries** |  |  |
| **Canada** | 2 | 50.0 |
| **Poland** | 2 | 50.0 |
| **others** | 0 | 0 |
| **Reporter** |  |  |
| **Consumer** | 0 | 0 |
| **Physician** | 1 | 25.0 |
| **Health Professional** | 2 | 50.0 |
| **Pharmacist** | 0 | 0 |
| **Not specified** | 1 | 25.0 |
| **Year of report** |  |  |
| **2004** | 0 | 0 |
| **2005** | 0 | 0 |
| **2006** | 0 | 0 |
| **2007** | 0 | 0 |
| **2008** | 0 | 0 |
| **2009** | 0 | 0 |
| **2010** | 0 | 0 |
| **2011** | 0 | 0 |
| **2012** | 2 | 50 |
| **2013** | 0 | 0 |
| **2014** | 0 | 0 |
| **2015** | 0 | 0 |
| **2016** | 0 | 0 |
| **2017** | 0 | 0 |
| **2018** | 0 | 0 |
| **2019** | 0 | 0 |
| **2020** | 0 | 0 |
| **2021** | 0 | 0 |
| **2022** | 0 | 0 |
| **2023** | 2 | 50.0 |
| **2024** | 0 | 0 |
| **2025** | 0 | 0 |
| **Outcomes** |  |  |
| **Death** | 0 | 0 |
| **Other serious outcome** | 2 | 50.0 |
| **Hospitalization** | 2 | 50.0 |
| **Life-Threatening** | 0 | 0 |
| **Disability** | 0 | 0 |

**Supplementary Table 22:**

**Clinical Characteristics of Depression-Related AEs in Patients Treated with Meropenem from the FAERS Database.**

| **Characteristics** | **Case number** | **Proportion (%)** |
| --- | --- | --- |
| **Number of events** | 3 |  |
| **Gender (%)** |  |  |
| **Female** | 2 | 66.7 |
| **Male** | 1 | 33.3 |
| **Not specified** | 0 | 0 |
| **Weight (kg)** |  |  |
| **<50** | 1 | 33.3 |
| **>100** | 0 | 0 |
| **50∼100** | 1 | 33.3 |
| **Not specified** | 1 | 33.3 |
| **Age(years)** |  |  |
| **<18** | 1 | 33.3 |
| **18∼65** | 1 | 33.3 |
| **65∼85** | 1 | 33.3 |
| **＞85** | 0 | 0 |
| **Not specified** | 0 | 0 |
| **Reported Countries** |  |  |
| **Sweden** | 1 | 33.3 |
| **China** | 1 | 33.3 |
| **The United States** | 1 | 33.3 |
| **others** | 0 | 0 |
| **Reporter** |  |  |
| **Consumer** | 0 | 0 |
| **Physician** | 1 | 33.3 |
| **Health Professional** | 0 | 0 |
| **Pharmacist** | 2 | 66.7 |
| **Not specified** | 0 | 0 |
| **Year of report** |  |  |
| **2004** | 0 | 0 |
| **2005** | 0 | 0 |
| **2006** | 0 | 0 |
| **2007** | 0 | 0 |
| **2008** | 0 | 0 |
| **2009** | 0 | 0 |
| **2010** | 0 | 0 |
| **2011** | 0 | 0 |
| **2012** | 0 | 0 |
| **2013** | 0 | 0 |
| **2014** | 1 | 33.3 |
| **2015** | 0 | 0 |
| **2016** | 0 | 0 |
| **2017** | 0 | 0 |
| **2018** | 0 | 0 |
| **2019** | 0 | 0 |
| **2020** | 1 | 33.3 |
| **2021** | 0 | 0 |
| **2022** | 1 | 33.3 |
| **2023** | 0 | 0 |
| **2024** | 0 | 0 |
| **2025** | 0 | 0 |
| **Outcomes** |  |  |
| **Death** | 1 | 33.3 |
| **Other serious outcome** | 1 | 33.3 |
| **Hospitalization** | 1 | 33.3 |
| **Life-Threatening** | 0 | 0 |
| **Disability** | 0 | 0 |

**Supplementary Figure 1:**

**Supplementary Figure 2:**
